# Development of a Phage Display Panning Strategy Utilizing Crude Antigens: Isolation of MERS-CoV Nucleoprotein human antibodies

**DOI:** 10.1038/s41598-019-42628-6

**Published:** 2019-04-15

**Authors:** Chia Chiu Lim, Patrick C. Y. Woo, Theam Soon Lim

**Affiliations:** 1 Institute for Research in Molecular Medicine, Universiti Sains Malaysia, 11800 Penang, Malaysia; 2 Department of Microbiology, The University of Hong Kong, Hong Kong SAR, People’s Republic of China; 3 Analytical Biochemistry Research Centre, Universiti Sains Malaysia, 11800 Penang, Malaysia

## Abstract

Antibody phage display has been pivotal in the quest to generate human monoclonal antibodies for biomedical and research applications. Target antigen preparation is a main bottleneck associated with the panning process. This includes production complexity, downstream purification, quality and yield. In many instances, purified antigens are preferred for panning but this may not be possible for certain difficult target antigens. Here, we describe an improved procedure of affinity selection against crude or non-purified antigen by saturation of non-binders with blocking agents to promote positive binder enrichment termed as *Yin-Yang* panning. A naïve human scFv library with kappa light chain repertoire with a library size of 10^9^ was developed. The improved *Yin-Yang* biopanning process was able to enrich monoclonal antibodies specific to the MERS-CoV nucleoprotein. Three unique monoclonal antibodies were isolated in the process. The *Yin-Yang* biopanning method highlights the possibility of utilizing crude antigens for the isolation of monoclonal antibodies by phage display.

## Introduction

Phage display technology is regarded as an important tool used for the discovery of novel ligands against various targets of interest^1^. It has since been widely employed for the *in vitro* development of monoclonal antibodies, trading places with the conventional hybridoma technology which was still mainly animal host dependent^2,3^. A detailed review on antibody phage display is provided by Ponsel, *et al*.^4^, Tohidkia, *et al*.^5^ and Zhao, *et al*.^6^. To identify a target specific antibody, an *in vitro* selection process underlined by the affinity enrichment concept known as biopanning is commonly used^2^. The affinity enrichment process involves several steps: immobilization of target antigen, binding of phage to target antigen, elimination of unbound phage and elution of phage^7^. The target antigens used for biopanning includes recombinant proteins, peptides, tissues and whole cells^8,9^. The main assurance is that these proteins are mainly prepared with good purity and yield. This is evident with the reports of antibody development for dengue virus^10^, herpes simplex virus^11^, cytomegalovirus^11^, Zika virus^12^, Ebola virus^7,13^, *Plasmodium* species^14^, *Mycobacterium tuberculosis*^15^, and nematodes responsible for filariasis^16^.

Nevertheless, several complications can be anticipated during antigen expression that hinders the availability of a target antigen for biopanning. Initially, solubility of the antigen is governed by the secretion of antigen into the cytoplasm (soluble form), periplasm (soluble form) and as inclusion body (insoluble form due to protein aggregation). Soluble proteins are desirable for protein purification whilst insoluble proteins must be solubilized, purified and re-folded. To overcome this issue, one has to go through a vigorous optimization process which includes cultivation temperature and conditions, expression using different *E. coli* expression hosts, co-expression with molecular chaperones, folding modulators and fusion partner proteins. A detailed explanation of recombinant protein expression has been reviewed by Gupta and Shukla^17^ and Rosano and Ceccarelli^18^. On top of that, another obstacle in the quest for recombinant protein production is the purification process. Protein purification from crude extracts using chromatography approaches caters to different properties of the protein. A common approach to capture the desired protein independent of unwanted proteins is by using affinity tags like His6x and glutathione-S-transferase to facilitate the purification process^19–21^. Even so, the purification scheme also requires tedious optimization in order to acquire a product of highest purity. All in all, the recombinant antigen has to be pure and abundant before it can be used for biopanning. Hence, this presents a major limitation when attempting to develop antibodies against challenging target antigens.

**Figure 1:**
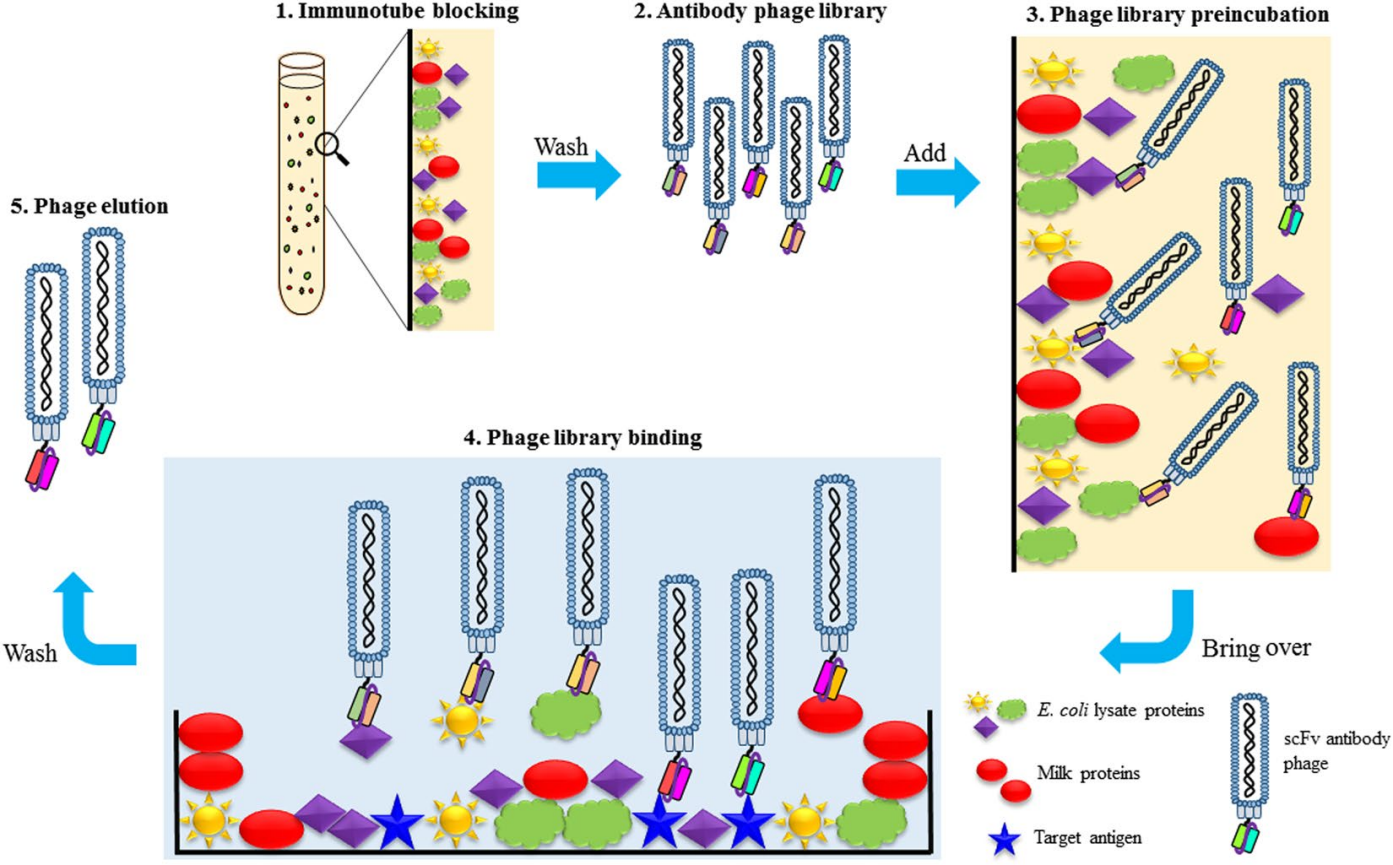
Schematic diagram of *Yin-Yang* biopanning method. The procedure involves the following steps, 1. Immunotube blocking with *E. coli* lysate and skimmed milk overnight, wash before use; 2. Adding the desired amount of antibody phage library into the immunotube; 3. Phage library preincubation takes place for 1 h in the immunotube; 4. Bring over the preincubated phage library into antigen-coated well for binding; 5. Stringent washing and elution of phage with trypsin enzymatic action.

In light of this, the establishment of a viable procedure for the selection of a desired antigen in crude preparation is of major interest. Herein, we propose a proof of concept panning process coined ‘*Yin-Yang*’ panning using antigen preparations devoid of purification for biopanning. We applied the process to isolate monoclonal scFv against the Middle East respiratory syndrome coronavirus (MERS-CoV) nucleoprotein (MERS-NP). The ‘*Yin-Yang’* concept takes its name from the Taoist concept of duality in striking a balance to form a whole. Here, the crude extract consisting of the antigen of interest in its native form alongside the endogenous *E. coli* proteins are used to achieve a balance between positive and negative selection. Due to the complex microenvironment during target enrichment, reducing non-specific binding interaction to *E. coli* proteins and blocking agents is paramount to allow effective antigen-antibody interaction to take place. A capture and elimination step was introduced prior to biopanning to remove the influence of binders against the non-target proteins. The complete workflow is outlined in the following schematic diagram (Fig. 1). The proposed approach was able to isolate monoclonal scFv clones against the MERS-NP utilizing a crude preparation. The antibodies were verified and identified for specificity against MERS-NP. This approach highlights the possibility of carrying out antibody phage display biopanning using crude antigens to identify antigen specific monoclonal antibodies.

## Results

### Antigen preparation

**Figure 2:**
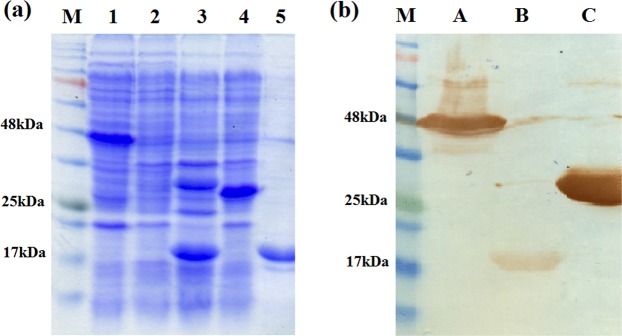
Coomassie stained SDS-PAGE of rMERS-NP, rUbi and *E. coli* lysates (**a**) and Western Blotting (**b**). M indicates BLUelf prestained protein ladder (GeneDireX Inc.); lane 1 and lane A, crude rMERS-NP; lane 2, *E. coli* C41 lysate; lane 3, crude rUbi; lane 4, *E. coli* BL21 with pRARE3 lysate; lane 5 and lane B, purified rUbi; lane C, positive control. The full length SDS-PAGE gel and blot are presented in Supplementary Figures 5 and 6.

The recombinant ubiquitin (rUbi) protein and its complementary *E. coli* lysate were prepared for the biopanning simulations while the rMERS-NP and its complementary *E. coli* lysate were produced for ‘*Yin-Yang*’ biopanning. The rUbi and rMERS-NP as well as their complementary *E. coli* lysates were successfully expressed utilizing similar host strains. The rUbi and rMERS-NP preparations were extracted and analysed with 12% SDS-PAGE. The crude extracts showed the presence of the other non-target proteins with a similar profile to the antigen preparations (Fig. 2a). The overexpression of rUbi and rMERS-NP were also visible in their respective crude preparations as a thicker band. Purification of the rUbi was carried out and the presence of the antigens were confirmed via Western Blotting using the His_6_-tagged secondary antibodies (Fig. 2b). The rUbi showed a specific band at the expected size of ~17 kDa with good yield and purity. The expected band size for rMERS-NP protein was ~46 kDa.

### The blocking effect of PTM buffer and *E. coli* lysate towards αUbi and M13KO7 phages against rUbi

**Figure 3:**
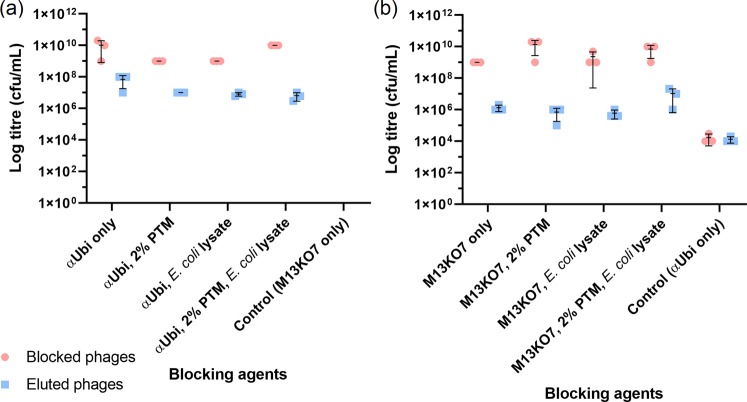
Phage titres of biopanning simulations of αUbi and M13KO7 incubated with various blocking agents. αUbi and M13KO7 were separately incubated in four blocking agents (PBS, 2% PTM, *E. coli* lysate, 2% PTM and *E. coli* lysate) during binding towards purified rUbi. The phages were collected at the end of binding by trypsin elution. αUbi (left) and M13KO7 (right) were collected before and after elution as ‘Blocked phages’ and ‘Eluted phages’. The data of mean ± s.d. (n = 3) of triplicates is shown.

Initially, a proof of concept was conducted to investigate the binding efficiency of the anti-Ubiquitin (αUbi) scFv phage towards rUbi in the complex microenvironment during biopanning. A total of four different conditions (complex microenvironment) were tested stepwise. First, both αUbi and M13KO7 phages were assimilated into different blocking agents: PBS, 2% PTM buffer, *E. coli* lysate and combinations of 2% PTM buffer with *E. coli* lysate. Phage titres were determined after binding between αUbi to rUbi and M13KO7 phage to rUbi. The unbound phages were collected as ‘Blocked phages’ and bound phages as ‘Eluted phages’ (Fig. 3). Based on the phage titres, αUbi phage showed good binding capacity towards rUbi in the presence of 2% PTM and *E. coli* lysate. Some amount of M13KO7 phage was also rescued despite incubation in different sets of blocking agents. Nevertheless, it should be highlighted that the combination of PTM buffer and lysate managed to block a large amount of αUbi and M13KO7 phage in separate sample sets. The extent of blocking was further determined by calculating the ratios of the difference between blocked αUbi in a mixture of PTM buffer and lysate against other sets of blocking agents. There was approximately a 10-fold difference between αUbi blocked with a combination of PTM buffer and lysate against the PTM buffer only and lysate only. A 0.83-fold difference was recorded between the PTM buffer and lysate combination against PBS only, where the cut off was 1-fold. The similar ratios of difference were determined for blocked M13KO7 sample sets at a cut off of 1-fold. The amount of blocked M13KO7 in the PTM buffer and lysate combination was higher by 7-fold with respect to M13KO7 in PBS only, 6.73-fold for M13KO7 in lysate only and 0.67-fold for M13KO7 in PTM only. It showed that the combination of PTM buffer and lysate was able to capture more unbound phages than other blocking agents.

**Figure 4:**
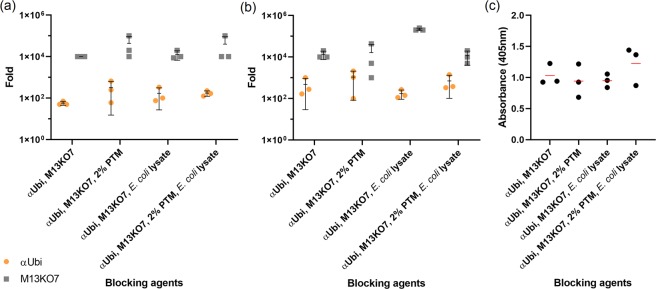
Phage titres of biopanning simulations with a mixture of αUbi and M13KO7 against purified rUbi (**a**) and crude rUbi (**b**) in the presence of PTM buffer and lysate. The mixture of αUbi and M13KO7 in 1:10 ratio was used to conduct biopanning simulations against purified rUbi and crude rUbi. The ratio of difference (fold change) were determined between the unbound phages and bound phages. (**a**,**b**) The fold change results are represented as mean ± s.d. (n = 3) as shown in the scatter dot plot. (**c**) Phage ELISA assay of biopanning simulation against crude rUbi was performed in triplicates and the absorbance for each sample were represented as mean (n = 3) (red line) after background removal. The overall s.d. for each triplicate was 0.17, 0.27, 0.11 and 0.31.

Next, a single round biopanning simulation was conducted in a similar fashion to further investigate the binding capacity of αUbi phage towards rUbi. αUbi and M13KO7 phage were mixed at a ratio of 1:10 and was used for biopanning simulation. The phage titres across 4 samples were determined as 10^8^–10^9^ cfu/mL, 10^6^–10^7^ cfu/mL, 10^8^–10^9^ cfu/mL and 10^4^–10^5^ cfu/mL for ‘Blocked αUbi’, ‘Eluted αUbi’, ‘Blocked M13KO7’ and ‘Eluted M13KO7’, respectively (Supplementary Fig. S1a). The binding between αUbi phage towards rUbi was relatively good despite a higher amount of M13KO7 was present in the sample. The ratios of difference between unbound and eluted fractions for αUbi phage across 4 samples were 57-fold, 325-fold, 169-fold and 172-fold (Fig. 4a). The fold change of unbound and eluted M13KO7 across 4 samples were large (10,000-fold, 43,333-fold, 12,916-fold and 40,000-fold). The highest ratio of difference was presented by M13KO7 blocked in PTM buffer, followed by a combination of PTM buffer and lysate and lysate only. This implied that the blocking agents were able to capture the background phages and reduce non-specific binding towards the antigen.

Lastly, crude rUbi was used as the target antigen in a similar biopanning simulation as mentioned above. Collectively, the phage titres for unbound αUbi phage (10^9^ cfu/mL) and unbound M13KO7 (10^9^–10^10^ cfu/mL) were slightly higher (about 1–3-fold for unbound αUbi phage and 5–13-fold for unbound M13KO7) than those compared to previous simulations using the purified rUbi fraction as shown in Supplementary Fig. S1b. On the other hand, the differences between the eluted αUbi phage with eluted M13KO7 were lower compared to the previous simulation. It was 55–180-fold in differences between the eluted αUbi phage with eluted M13KO7 for previous simulation whereas the differences dropped to only 2–5-fold, with the exception for sample 3 (300-fold). This was due to the abrupt drop of eluted M13KO7. The binding capacity of αUbi phage towards rUbi in crude fraction was consistent with previous simulations, as the similar amount of eluted αUbi phages was determined to be in the range of 10^6^–10^7^ cfu/mL.

The highest ratio of difference between unbound and eluted αUbi phage was recorded from sample 2 (PTM buffer only) with a 1,000-fold difference followed by a 700-fold difference recorded from sample 4 (PTM buffer and lysate). As for the ratios of difference between unbound and bound M13KO7, the highest fold change was 210,000-fold recorded from sample 3 (lysate only) while the lowest fold change was determined from sample 4 (PTM buffer and lysate) with only 11,000-fold difference (Fig. 4b). There were different trends exhibited from the fold change between unbound and bound αUbi phage with M13KO7 but the blocking effects of PTM buffer and lysate were able to be identified. The phage ELISA was conducted to measure the performances and effects of different blocking agents on the binding of αUbi phages against crude rUbi. Out of the four samples, the combination of PTM buffer and lysate showed the highest signal (1.226) detected with anti-c-myc-HRP as shown in Fig. 4c. This combination of blocking agents was able to control the interference of background phages without compromising the binding capacity of αUbi phage. Therefore, this combination of blocking agents was used for the next stage of biopanning optimization.

### Lysate preblocking and phage preincubation effects towards αUbi and M13KO7 phages against rUbi

**Figure 5:**
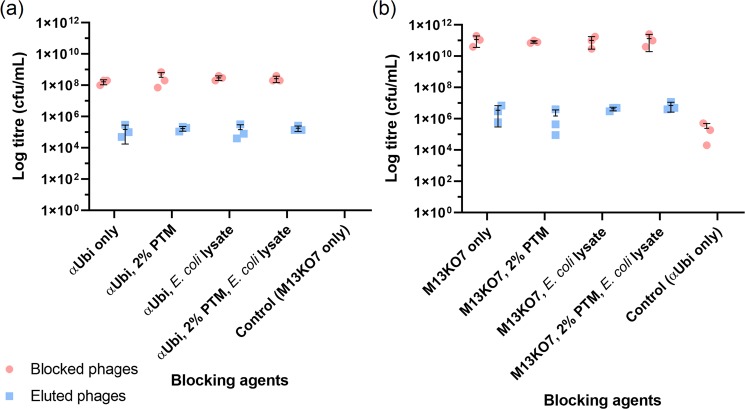
Phage titres of biopanning simulations of αUbi and M13KO7 with lysate preblocking and phage preincubation. αUbi and M13KO7 were separately incubated in the lysate/PTM-preblocked microwells in four different blocking agents (PBS, 2% PTM, *E. coli* lysate, 2% PTM and *E. coli* lysate). At the end of preincubation, the phages were transferred to antigen-coated wells for binding at 37 °C, 600 rpm for 2 hours. αUbi (left) and M13KO7 (right) were collected before and after elution as ‘Blocked phages’ and ‘Eluted phages’. The data shows the mean ± s.d. (n = 3) of triplicates.

To further optimize the biopanning conditions against crude antigens, the *‘Yin-Yang’* (capture and eliminate) approach was designed to reduce non-specific binding by background phages. The approach utilized *E. coli* lysate and PTM to block the microwells, followed by incubation of phages with PTM buffer in the prepared microwells. This procedure imposes a capture and elimination effect to the phage mixture, enabling non-specific phages to be preoccupied from interfering in the binding activity. Initially, αUbi and M13KO7 phages were subjected to binding with purified rUbi. The unbound phages were collected as ‘Blocked phages’ and bound phages as ‘Eluted phages’ (Fig. 5). The overall phage titres for unbound M13KO7 were higher compared to the previous optimization without lysate preblocking and phage preincubation by approximately 25 to 100-fold. Also, the differences of unbound and bound M13KO7 were larger compared to the last optimization by more than 10-fold. Therefore, lysate preblocking and phage preincubation was found to reduce the non-specific binding of background phage more effectively.

**Figure 6:**
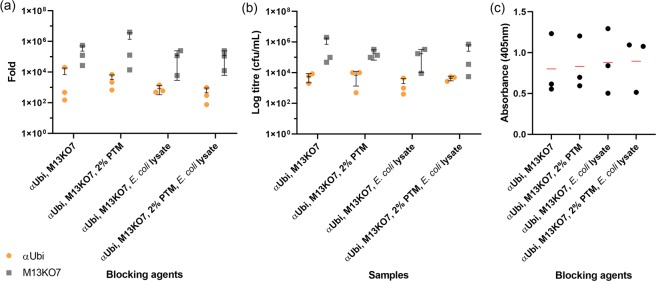
Phage titres and phage ELISA of ‘*Yin-Yang*’ biopanning simulation with a mixture of αUbi and M13KO7 against purified rUbi (**a**) and crude rUbi (**b**). The mixture of αUbi and M13KO7 in 1:10 ratio was used to conduct ‘*Yin-Yang*’ biopanning simulations against purified rUbi and crude rUbi. The ratio of difference (fold change) were determined between the unbound phages and bound phages. (**a**,**b**) The fold change results are represented as mean ± s.d. (n = 3) as shown in the scatter dot plot. (**c**) Phage ELISA assay on ‘*Yin-Yang*’ biopanning simulation against crude rUbi was performed in triplicates and the absorbance for each sample were represented in mean (n = 3) (red line) after background normalization. The overall s.d. for each triplicate was 0.38, 0.33, 0.40 and 0.33.

Next, a mixture of αUbi and M13KO7 (1:10 ratio) was subjected to incubation with PTM buffer in lysate/PTM-preblocked microwell (‘*Yin’* step) prior to binding with purified rUbi (‘*Yang’* step). A higher number of unbound M13KO7 phage at 10^10^ cfu/mL was rescued or determined (Supplementary Fig. S2a). This was a 10-fold increase in comparison to the biopanning process devoid of preblocking and preincubation, indicating that the *Yin-Yang* process was more efficient in removing non-specific phage particles. However, higher numbers of eluted M13KO7 phage particles were also obtained indicating more background phages were recovered during the simulation. Nevertheless, the bound αUbi phage was slightly higher than the bound M13KO7 by 4–32-fold. The ratio of difference between unbound and bound αUbi phage was determined (Fig. 6a). The highest difference was recorded from sample 1 (PBS only) at 6,800-fold whereas the lowest difference was sample 4 (PTM buffer and lysate) at only 450-fold. The highest fold change between the unbound and bound M13KO7 was determined from sample 2 (PTM buffer) at 1380000-fold, followed by sample 3 (lysate) and sample 4 (PTM buffer and lysate), both were 125,000-fold. This showed that PTM buffer and lysate were suitable for preblocking and phage preincubation.

Lastly, biopanning was conducted using crude rUbi as the target antigen. Based on the phage titres in Supplementary Fig. S2b, the amount of unbound M13KO7 (10^10^ cfu/mL) were similar to that of the biopanning simulation using purified rUbi. This indicates the efficiency of *E. coli* lysate and PTM buffer blocking to remove non-specific interaction from crude fractions. As for bound phages, the number of bound M13KO7 (10^4^–10^6^ cfu/mL) however was almost the same as the bound αUbi phages (10^4^–10^5^ cfu/mL) with a difference of only 0.5-4-fold. This pattern was expected, as the difference of bound phages in previous simulation (with pure rUbi) was also minimal. Notably, the amount of bound M13KO7 for sample 4 (PTM buffer and lysate) was 10-fold higher than αUbi phage. This showed a dominant population of empty phages in the eluted phages rather than antibody presenting phages. The highest fold change between unbound and bound αUbi phage was recorded from sample 2 (PTM only) at 6,800-fold, followed by sample 1 (PBS only) (5,500-fold) and sample 4 (PTM and lysate) (4,300-fold) (Fig. 6b). On the other hand, the highest ratio of difference between unbound and bound M13KO7 was determined from sample 1 (PBS only) at 716,000-fold, followed by the PTM buffer and lysate combination (254,000-fold). This implied that the *‘Yin’* step was sufficient to deplete the non-specific binding of M13KO7. Moreover, the binding capacity of αUbi phage towards crude rUbi was not affected by the *‘Yin’* effect, but in contrast helped to control the interference of background phages.

The eluted phages were amplified and packaged for the subsequent phage ELISA to assess the ‘*Yin-Yang’* effect utilising different blocking agents. Out of the four samples, the combination of PTM buffer and lysate showed the highest signal (0.895) detected with anti-c-myc HRP as shown in Fig. 6c. Despite a drop in the binding of αUbi towards the crude rUbi that was evident with a lower OD_405nm_ readout, it could be concluded that the ‘*Yin*’ effect is essential and the use of the PTM buffer and lysate combination was suitable to reduce the non-specific interaction as designed by the ‘*Yin*’ effect.

### Human naive kappa light chain scFv phagemid library construction

A naïve scFv library was constructed using healthy human samples focusing on the IgM V_H_ and V_K_ repertoire. The diversity of the constructed naïve antibody phagemid library was estimated to be 10^9^. Colony PCR was performed and resolved by agarose electrophoresis as shown in Supplementary Fig. S3. A total of 7 colonies from 10 randomly selected colonies presented a single band of the expected amplicon size at approximately 1,100 bp. This indicated that full-length scFv constructs were incorporated into the phagemid vector at a reasonable success rate of 70%.

### *‘Yin-Yang’* biopanning and polyclonal ELISA

The in-house human naïve kappa light chain scFv library was subjected to *‘Yin-Yang’* biopanning against crude rMERS-NP. A higher percentage of blocking agent and an increase in washing stringency were introduced during the biopanning rounds based on the conditions from the earlier optimization steps. In just two rounds of affinity selection, there was an enrichment of specific anti-rMERS-NP antibodies. Supplementary Fig. S4 shows the polyclonal ELISA result of crude rMERS-NP biopanning at absorbance 405 nm. A_405nm_ were slightly low at 0.05 and 0.17 for the first and second round of biopanning respectively. This could be attributed to the low amount of rMERS-NP being presented in the total crude lysate. The phage titre determination indicates approximately 2.5 folds of enrichment was observed. The phage enrichment was tabulated in Supplementary Table S1 that complements the polyclonal ELISA result.

### Monoclonal eLISA

**Figure 7:**
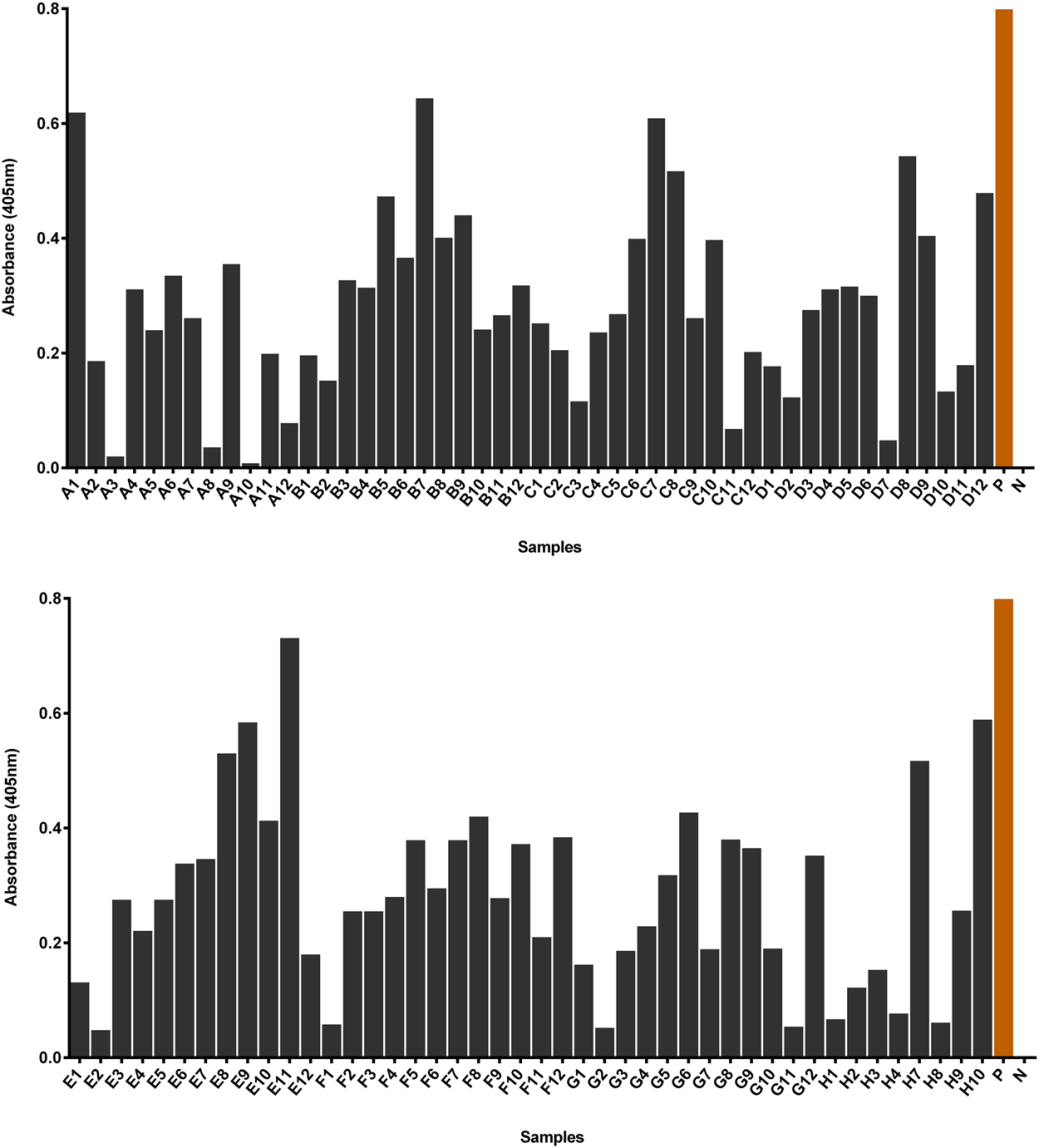
Histogram showing the absorbance of monoclonal scFv antibody phage binding against crude rMERS-NP after normalizing with background values. H5 and H11 are the positive controls, labelled as P (OD_405nm_ readouts 3.54 and 3.90, respectively) while H6 and H12 are the negative controls, labelled as N (OD_405nm_ readouts 0).

A total of 92 individual clones were selected from the second polyclonal pool for monoclonal ELISA assay against crude rMERS-NP. The positive clones were identified with a cut-off OD_405nm_ value above 0.3 after normalizing with their background values. The readouts of positive controls in the monoclonal ELISA were 3.5 and 3.9 respectively (H5 and H11, labelled as P), while the negative controls were below 0.1 (H6 and H12, labelled as N) (Fig. 7). Overall, the monoclonal ELISA assay resulted in 40 positive binders. The generated human naïve library was able to provide satisfactory enrichment for anti-rMERS-NP. A preliminary screening by colony PCR was subjected to these 40 positive clones to identify clones with full-length inserts before identification by sequencing.

### Sequence analysis

Sequencing results of the selected binders were analysed by IMGT/V-QUEST to determine the identity of the scFv monoclonal antibody clones. Only 3 full length antibody clones were obtained that showed unique combinations of heavy chain and kappa light chain families. In addition, 2 clones were found to have deletions at their gene segments but not at the complementarity-determining regions (CDRs) and were categorized as partial clones. Then, 3 clones presented with umber and ochre stop codons while 2 truncated clones were also obtained from the anti-rMERS-NP pool. A compilation of the gene segment usage for the full clones is presented in Table 1.

**Table 1:** Antibody sequence analysis of the anti-rMERS-NP monoclones.

Binder name	V-gene family	D-gene family	J-gene family
A4	IGHV4-34*01	IGHD7-27*01	IGHJ4*02
	IGKV2D-29*01	—	IGKJ1*01
C8	IGHV1-69*01/69D*01	IGHD6-6*01	IGHJ6*02
	IGKV2-28*01/2D-28*01	—	IGKJ2*01
G12	IGHV3-13*01	IGHD6-19*01	IGHJ2*01
	IGKV1-39*01/1D-39*01	—	IGKJ2*01

### Preparation of antibody fragments

**Figure 8:**
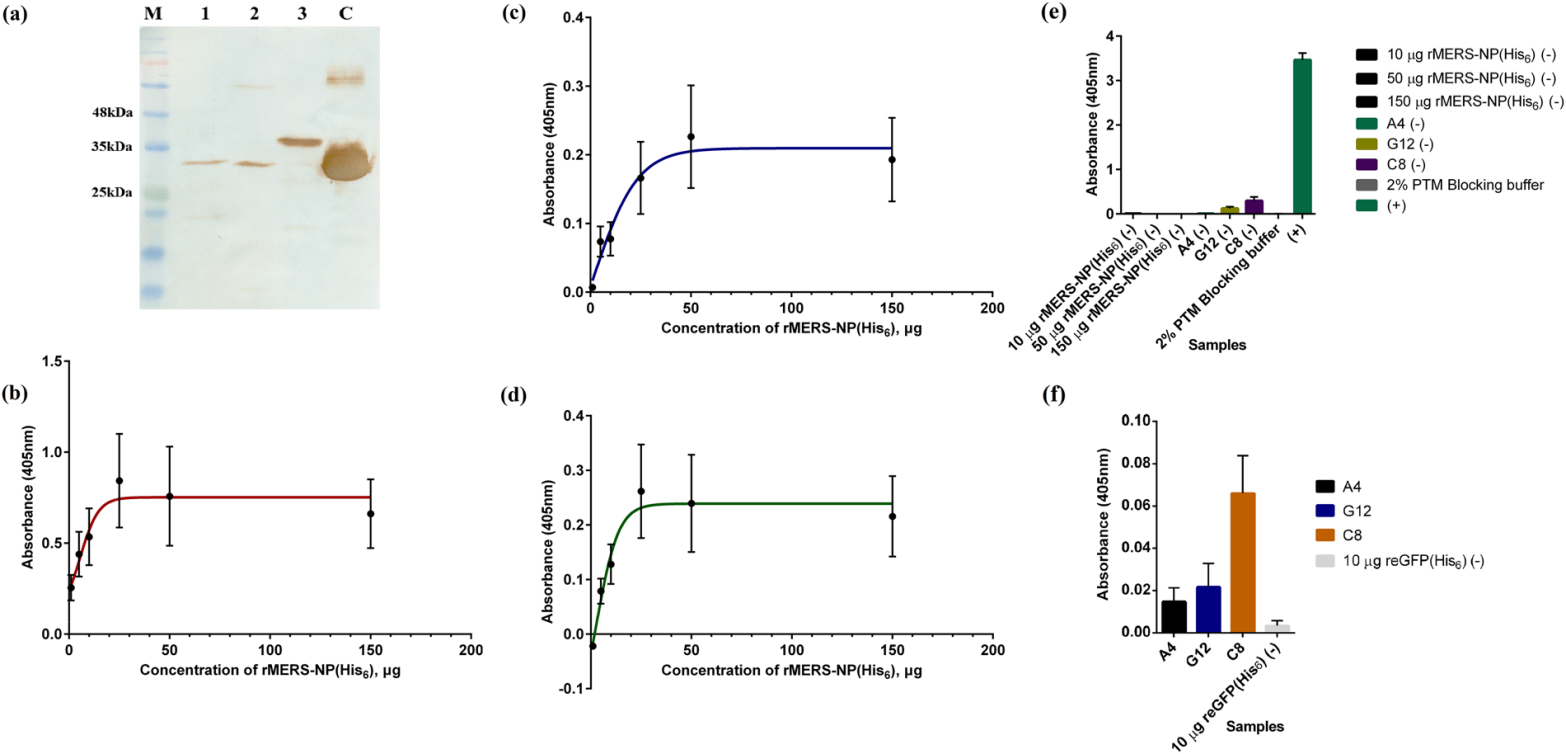
The production of antibody fragments and their antigen binding ELISA assay against rMERS-NP(His_6_) and cross-reactivity assay against other His_6_-tagged protein. (**a**) Western blotting analysis of rA4scFv, rG12scFv and rC8scFv using streptavidin-HRP was performed. M indicates Opti-Protein XL Marker prestained protein ladder (Abm Inc.); lane 1, purified rA4scFv; lane 2, purified rG12scFv; lane 3, purified rC8scFv; lane C, positive control. (**b**–**e**) The antibody fragments were subjected to antigen binding ELISA assay against a serial dilution of rMERS-NP(His_6_) at various concentrations. The absorbance of each set of samples after normalizing with background values are presented as mean ± s.d. (n = 3) in the line graphs, (**b**) rA4scFv, (**c**) rG12scFv and (**d**) rC8scFv. (**e**) is the absorbance of the control set during the ELISA assay. (**f**) Cross-reactivity ELISA assay of antibody fragments against other His_6_-tagged protein was also performed. The control antigen used is 10 μg of rGFP(His_6_). The absorbance for all sample sets were collected in triplicates and presented as mean ± s.d. (n = 3) in the bar graph.

The clones A4, C8 and G12 were successfully expressed as inclusion bodies and subjected to solubilization using 8 M urea buffer (pH 8). Western Blotting using streptavidin-HRP was performed to detect the biotinylated antibody fragments as shown in Fig. 8a. The purified recombinant antibody fragments showed a distinct band at the expected size of ~32 kDA for rA4scFv and rG12scFv. Despite the expected size of rC8scFv is ~32 kDa, it ran slightly higher at ~35 kDa.

### Antigen binding ELISA

The preparation of purified rMERS-NP(His_6_) and rGFP(His_6_) for ELISA were detailed in the Supplementary Method 10. The expression profiles of the antigens were included in Supplementary Result 7. The binding profiles of the recombinant antibody fragments were determined against a range of rMERS-NP(His_6_) from 1 μg to 150 μg. Each sample set was incubated with 10 μg of purified antibody. In order to assess the cross-reactivity of the antibody towards other His_6_-tagged proteins, rGFP(His_6_) was used. A total of 10 μg of purified rGFP(His_6_) was coated and allowed to bind with 10 μg of each antibody clone respectively. Figure 8b–d show the binding profiles of the respective antibody fragments towards rMERS-NP at A_405 nm_ after normalizing with their background values and controls (Fig. 8e).

Overall, the binding performance of rA4scFv was the best in a dilution series of rMERS-NP(His_6_) concentration with the highest absorbance values. The interactions of rA4scFv and rC8scFv towards rMERS-NP(His_6_) peaked at a concentration of 25 μg (OD_405nm_ 0.843 and 0.262) while binding of rG12scFv peaked at a concentration of 50 μg of rMERS-NP(His_6_) with OD_405nm_ readout 0.226. The binding of rA4scFv and rC8scFv towards rMERS-NP(His_6_) has reached an apparent saturation at a concentration of 30–50 μg antigen while rG12scFv reached the apparent saturation point at the beginning of 50 μg of rMERS-NP(His_6_). The background values of rG12scFv and rC8scFv were slightly higher (0.193 and 0.216 respectively), therefore giving low OD_405nm_ readouts after normalization. The cross-reactivity profiles of the antibody fragments towards rGFP(His_6_) at absorbance 405nm after normalizing with their background values are shown in Fig. 8f. The data also takes into consideration cross reaction against streptavidin-HRP. Overall, the antibody fragments had very little binding towards the control antigen as the readouts were lower than 0.1. Therefore, the antibody fragments could be identified as specific towards the rMERS-NP.

## Discussion

To date, antibody phage display still persist as the method commonly used for the generation of recombinant antibodies against various targets for biomedical applications and research^6,22^. The robustness of bacteriophage to display antibodies has aided to expand the portfolio of target antigens which includes toxins, transmembrane proteins, peptides, haptens and even epitopes^23^. Unfortunately, the problems of expression and purification of *E. coli* produced recombinant proteins are atypical. Some recombinant antigens have solubility and purity problems^18^, stability issues, difficult to be immobilized^24^ and exist in multiple morphologies^25^. Due to these reasons, there is a need to develop an alternative procedure for selection towards relevant antigens presented in a complex mixture especially in situations when rapid antibody development is required or if the purified target is unavailable. Therefore, we demonstrated a capture and elimination step prior to affinity selection in order to provide a ‘subtractive’ effect to the phage population by blocking the undesired binders via binding to other components in the blocking mixture while freeing the positive binders to favour binding towards the relevant antigen.

The’*Yin-Yang’* panning method is somewhat akin to the negative/positive selection panning strategies whereby negative selection aims to remove unwanted binders and allowing positive selection of targeted binders^24,26,27^. The main difference between this study and the previous methods is the approach and target of negative selection. The target for negative selection is the unspecific antibody phage binders that binds to proteins other than the target antigen. In light of this, the blocking mixture is mainly used to deplete the negative binders. In the previous approaches, polyclonal and mAbs are used to target the unrelated epitopes presented on phages in order to select and remove them, allowing the remaining phage clones to select for antibodies that have affinity towards specific epitopes^24,26,27^. Moreover, the ‘*Yin-Yang*’ method offers two rounds of negative selection during a single round of biopanning, one before positive selection and one simultaneously carried out with the positive selection. This creates a competitive situation where a small number of unbound negative phage will compete with a large number of unbound positive phage for the target antigen in the presence of target-unrelated proteins. We demonstrated an inexpensive immobilisation strategy of target-unrelated proteins onto a solid phase for negative selection, whereby unrelated proteins preoccupy non-specific binders, either bound to surface or in phage mixture. The limitation of this method is the inability to remove the non-specific binders prior to binding. However, imposing a more stringent washing process during phage elution can compensate for this. Therefore, the phages binding to unspecific proteins can be washed away under stringent conditions, subsequently rescuing bound phages that is immobilised on the plate. Additionally, this method can also be adapted to membrane-bound proteins by using the western blotting system reported by Ding, *et al*.^28^ and Liu, *et al*.^29^. A comparison of other published enrichment strategies and the ‘*Yin-Yang’* method is provided in Table 2.

**Table 2:** Overview of the available enrichment strategies.

Method & Description	Ligand format	Enrichment fold/number of positives on mAb ELISA (%)	Targets				Reference
			Purified antigen	Complex antigen	Transmembrane proteins/cell surface receptors	Cell	
*Subtractive selection* Eliminating cells that do not express target proteins prior to selection	scFv	Targeted cells/leukocytes, ~3.5 fold	N/A	N/A	+	+	de Kruif, *et al*.^64^
*Depletion selection ‘BRASIL’* Depleting non-antigen binders prior to selection	Peptide	vascular endothelial growth factor (VEGF), 2–3 fold	N/A	N/A	+	+	Giordano, *et al*.^65^
*Subtraction/depletion* Removing unwanted epitopes prior to selection	scFv	Glycoprotein IIb/IIIa, 2–5 fold	N/A	N/A	+	N/A	Eisenhardt, *et al*.^66^
*Microselection*/*shadow-stick selection* Targeting a single rare cell and ultra-violet (UV) inactivation of the non-binders	scFv	Targeted rare cells, 10–25%	N/A	N/A	+	+	Sørensen, *et al*.^67^ Sørensen M. D. and Kristensen P.^68^
*Masked selection* Using non-relevant soluble binders to block non-relevant epitopes prior to selection	VHH	Recombinant HER2-Fc Protein, 92.6%	+	N/A	N/A	N/A	Even-Desrumeaux, *et al*.^69^
		HER2 receptor, 26%	N/A	N/A	+	+	
		mGluR4 receptor, 90%	N/A	N/A	+	+	
		CXCR4 receptor, 24%	N/A	N/A	+	+	
		Breast Cancer Biopsy Lysates, 40%	N/A	+	+	+	
		Cell Surface Binders on Breast Cancer Cell Lines, 60%	N/A	N/A	+	+	
*Negative selection* Eliminating dead cells and cell debris prior to selection	scFv	61% intact cells recovered	+	N/A	+	+	Pavoni, *et al*.^70^
*Subtractive selection* Removing non-binders using non-transfected cells prior to selection	scFv	Membrane bound antigens, 4–5 fold	N/A	N/A	+	+	Jones, *et al*.^71^
*‘Yin-Yang’* Using target -unrelated proteins to preoccupy non-specific binders prior to selection	scFv	rMERS-NP, 2.5 fold/41.7%	+	+	N/A	N/A	This study

N/A = Non-applicable as the publications do not include information.

Preliminary optimization was performed in a stepwise manner to investigate the strength of blocking agents towards phage binding capabilities for both antibody phage and phage without antibody using αUbi and M13KO7. The use of αUbi as a model phage for optimization represents the specific rUbi positive binders while M13KO7 represents the population of non-specific binders. The mixture of PTM and *E. coli* lysate exerts the highest blocking effect for both αUbi and M13KO7. Nonetheless, the bulk M13KO7 population (about 10^11^ cfu/mL) is expected to saturate the sample thus allowing it to adsorb and bind non-specifically to rUbi which can be recovered later. M13KO7 binds at low affinity towards other antigens as reported by Lamboy *et al*.^30^ and modifications have been performed to reduce unspecific binding by M13KO7.

To simulate the similar conditions during affinity selection, αUbi and M13KO7 were mixed to represent a cocktail of library phage for panning against rUbi in purified and crude forms^31^. Competition is expected to occur between positive binders and non-specific binders against the target antigen. Thus, a competitive selection could be imposed by saturating the non-specific binders, M13KO7 with the competitive proteins (lysate and PTM) thereby promoting binding of αUbi towards purified rUbi^32^. Despite the presence of other unknown *E. coli* proteins in the crude fraction of rUbi with the actual concentration of rUbi being expected to be lower than the purified rUbi, the binding capability of αUbi is still unaffected as shown in phage ELISA assay upon affinity selection against crude rUbi. The blocking effect of PTM and *E. coli* lysate was the highest among the samples and was able to block M13KO7 from competing with αUbi. There was no bias in the titres of packaged phages for ELISA assay as they were normalized to 10^11^ before assayed.

During the *‘Yin-Yang’* (capture and elimination) step, preincubation of phage was performed in wells coated with *E. coli* lysate and subsequently with PTM. Proper blocking of the surface with blocking agent such as skimmed milk helps to inhibit unspecific binding^33–35^ and phage preincubation can enhance the reduction of unspecific binders even to the treated surface^36^. The idea of using *E. coli* lysate proteins as one of the competitive proteins in biopanning originates from the whole cell biopanning approach, where target antigen and un-relevant proteins are presented in the cell lysate mixture^33^. Theoretically, it could be expected that non-binders were depleted during the *‘Yin-Yang’* step, subsequently allowing free positive binders to initiate binding against the target during affinity selection. The *‘Yin-Yang’* step complements the blocking effect by the lysate and PTM, thereby allowing more non-binders to be trapped. This was evident as a higher number of unbound M13KO7 was recorded. However, throughout the biopanning simulation against rUbi in purified and crude forms, there were increasing numbers of non-binders being rescued, close to the amount of positive binders rescued. This could be attributed to insufficient washing that resulted in the rescue of non-binders together with positive binders during elution^37^. Despite this, the rescued phages from the crude rUbi were amplified and subjected to phage ELISA assay to determine the best performing sample. The best performing sample was the lysate and PTM combination that gave the highest readout after background subtraction. Hence, we demonstrated that the *‘Yin-Yang’* step is crucial during biopanning against crude antigen while the combination of PTM and lysate can help to control and reduce the disturbance of non-binders during antigen binding. Nevertheless, due to the rise of background phage elution, washing stringency should be revised accordingly. The rationale of coating the phage pre-incubation well with lysate and then with PTM is to inhibit the plastic binders from interfering in the optimization steps^38^ and for further negative selection.

In this work, a naïve human antibody phage library was generated for panning against the MERS-CoV nucleoprotein. The naïve library was constructed from 52 healthy donors comprising of three ethnic groups. This is aimed to expand the diversity of antibody repertoire for a highly diverse antibody library^39^. The ‘single pot’ antibody library repertoire has a universal characteristic, which makes it suitable to be employed for antibody selection against a wide range of target antigens^39,40^. To generate a library of highest possible diversity, size and quality, combinatorial mixing of heavy and light chains was performed to create all possible gene combinations. The V-gene repertoire was amplified using a gene specific primer set with a high coverage of antibody genes^5^. In addition to that, the genetic diversity from donor samples of three ethnic groups may offer additional sequence diversity. Cloning efficiency was improved by performing an intermediary cloning to ensure highest incorporation of full antibody gene sequences into the destination phagemid vector. The library generated has a modest diversity of 10^9^ that is sufficient for antibody selection. Despite naïve libraries have been reported to yield low affinity binders, it is still an important source for antibodies. A correlation between library diversity with antibody affinity was proposed to be directly proportional^41^. Even so, the low affinity binders can undergo *in vitro* affinity maturation to improve their binding affinity and specificity^5,39,42^. Kappa light chain isotype was solely used with the heavy chain IgM isotype repertoire for this naïve library as more kappa antibodies are reported in the human peripheral blood than lambda antibodies. This is because rearrangement of kappa isotypes occurs earlier but when both kappa alleles failed to rearrange productively, this is followed by lambda rearrangement^43^. However, this ratio changes upon encountering antigens, suggesting that light chain rearrangement occasionally takes place in B cell after the onset of somatic hypermutation^44^. This is in line with the structural and physiochemical studies of kappa and lambda antibodies, with both exhibiting differential characteristics during adaptive immune response^43,45^. However, in this context, we sought to generate a naïve library for kappa antibodies while further studies on differential properties of lambda and kappa antibodies will be carried out later.

In this study, the naïve library was subjected to the revised affinity selection to isolate antibodies against rMERS-NP from a crude preparation. *‘Yin-Yang’* biopanning was able to enrich antibodies against crude rMERS-NP after 2 rounds of selections by 2.5-fold. The low enrichment ratio was expected due to the loss of binders to interfering proteins from the crude fraction. The method is able to impose both the competitive and subtractive effect onto non-specific binders in order to increase the possibility of positive binders to bind the target in extremely low amounts. Reproducibility of this method is fairly good as good enrichment was achieved in several attempts using other in-house antibody libraries against different targets (data not shown). MAb selection was done and 3 full antibody clones were obtained for this work. Based on the sequence analysis, the V-gene combination of clone rMERS-NP-A4 is IGHV4-IGKV2, IGHV1-IGKV2 for rMERS-NP-C8 and IGHV3-IGKV1 for rMERS-NP-G12. The antibody fragments were expressed, purified and their binding specificity determined towards rMERS-NP. rMERS-NP-A4 binds specifically to rMERS-NP with very minimal background signal while rMERS-NP-C8 and rMERS-NP-G12 presented some degree of background signal indicating the clones as low performing clones. We noticed a slight difference in the antibody band migration which may likely be due to the introduction of chemical modifications^46^, which in this case is the biotinylation that contributed to the retardation of gel migration. In addition, previous reports examined the efficiency of biotinylation by comparing the gel migration of biotinylated products against non-biotinylated products in the presence of streptavidin and reveal a slower migrating of biotinylated products^47–51^. Despite all three antibody fragments underwent biotinylation, the gel shift effect was more drastic for rC8scFv at approximately 3–4 kDa difference in comparison to rA4scFv and rG12scFv.

Previously reported neutralizing mAbs against MERS-CoV were specific to the receptor-binding domain (RBD) of the spike (S) glycoprotein of MERS-CoV^52^. The RBD of MERS-CoV binds to human dipeptidyl peptidase 4 (hDPP4) (cellular receptor) and gains entry into the target cell for viral infection. Most of the reported mAbs has been focused on the neutralization of RBD to block the interaction of RBD to hDPP4 and extreme potency has been exhibited by mAbs targeting RBD. Despite hDPP4 is another potential target for neutralization, it is also a particularly important molecule in the immune regulation of T cell activation and chemokine function. Therefore, clinical use of it is unadvisable as adverse side effects may arise^53,54^. A recent report combined mAbs specific to RBD and non-RBD (other domains on S glycoprotein) to initiate neutralizing enhancement as different antigenic sites on S proteins are targeted^55^. Nevertheless, immune escape is always endeavoured by coronaviruses by expression of mutant S proteins while nucleocapsid proteins have less mutations. The abundant NP of MERS-CoV makes it another potential candidate target other than the S proteins. NPs are responsible for viral genomic RNA packaging, transcription and assembly^56^. In a previous report, three mAbs against MERS-CoV RBD with exceptional potency was found to originate from the IGHV1-69 heavy chain family^57^. Interestingly, in this study, we found that rMERS-NP-C8 clone targeting the NP also belongs to IGHV1-69 heavy chain family. Of note is that the IGHV1-69 gene was also found to be utilized by other antiviral antibodies targeting HIV-1, influenza virus and hepatitis C virus^54^.

This ‘*Yin-Yang’* method together with the naïve scFv library was able to select antibodies against a complex antigen by providing competitive and subtractive effects to favour binding of positive binders. This permits antigens in different states (complex mixture, low purity, protein cocktails) to gain excess for affinity binding without the limitations of protein purity and yield. It is likely that optimization of this approach is required for different antigens produced in different host cells due to the differences in cell contents. The generated kappa light chain scFv library was able to generate mAbs against MERS-CoV NP using the proposed approach with crude rMERS-NP. Therefore, this kappa exclusive naïve library is potentially useful for isolation of antibodies against other interesting antigens in the future. In conclusion, the *‘Yin-Yang’* panning together with the kappa naïve scFv library can be used as an alternative selection process for mAb generation against challenging target antigens.

## Methods

### Bacterial strains

*Escherichia coli* BL21 (DE3) F^−^ dcm ompT hsdSB(rB^−^ mB^−^) gal λ(DE3), *E. coli* C41 (DE3) F^−^ dcm ompT hsdSB (rB- mB-) gal (DE3) (Agilent Technologies, Santa Clara, CA, USA), *E. coli* Shuffle^®^ T7 F′ lac, pro, lacIq/Δ(ara-leu)7697 araD139 fhuA2 lacZ::T7 gene1 Δ(phoA)PvuII phoR ahpC* galE (or U) galK λatt::pNEB3-r1-cDsbC (SpecR, lacIq) ΔtrxB rpsL150(StrR) Δgor Δ(malF)3 (New England Biolabs (NEB), MA, USA), *E. coli* TG1 supE thi-1 ∆(lac-proAB) ∆(mcrB-hsdSM)5 (rK^−^ mK^−^) [F′ traD36 proAB lacIq Z∆M15] (Agilent Technologies, Santa Clara, CA, USA), *E. coli* XL1-Blue recA1 endA1 gyrA96 thi-1 hsdR17 supE44 relA1 lac [F′ proAB lacIq Z∆M15 Tn10 (Tetr)] (Agilent Technologies, Santa Clara, CA, USA) and *E. coli* TOP 10 F^-^ mcrA Δ(mrr-hsdRMS-mcrBC) Φ80lacZΔM15 Δ lacX74 recA1 araD139 Δ(araleu)7697 galU galK rpsL (StrR) endA1 nupG (Invitrogen, MA, USA).

### Plasmids

Helper plasmid pRARE3 encoding the biotin ligase and rare tRNAs as well as pRSET-BH6 plasmid were obtained from Dr. Zoltan Konthur, Max Planck Institute for Molecular Genetics (Berlin, Germany). pCR^™^ 2.1-TOPO^®^ vector was acquired from Invitrogen. A pLABEL (phagemid) vector^58^ was used to incorporate scFv fragments for phagemid library generation. rGFP and αUbi scFv were adapted from Ismail, N. F. *et al*.^59^.

### Phage packaging methods

The propagation and amplification as well as phage packaging were performed according to Hust and Mersmann^36^, Hust and Lim^60^ and Lim, *et al*.^61^.

### *E. coli* lysate, recombinant ubiquitin (rUbi) and recombinant MERS-nucleoprotein (rMERS-NP) preparation

To prepare rUbi and its complementary *E. coli* lysate, the recombinant plasmid pRSET-BH6 bearing the *ubi* gene and the empty plasmid with helper plasmid pRARE3 were introduced into *E. coli* BL21 (DE3) strain. The transformed cells were grown in 2YT media supplemented with 0.2% (v/v) glucose, 100 µg/mL ampicillin, 17 µg/mL chloramphenicol and 1 mM isopropyl β-D-1-thiogalactopyranoside (IPTG) at 25 °C for 16 h. The bacterial cell pellets were collected and subjected to cytoplasmic extraction with 20 mg/mL lysozyme followed by 4 min sonication on ice. IMAC purification was performed for rUbi using a 1 mL Ni-NTA Agarose fast flow column (GE Healthcare). The recombinant plasmid pRSET-BH6 bearing MERS-NP gene was expressed in a similar fashion using *E. coli* C41 (DE3) strain and followed by cytoplasmic extraction. The crude proteins and lysates were subjected to SDS-PAGE analysis.

### *‘Yin-Yang’* biopanning optimization: Blocking effects of PTM buffer and *E. coli* lysate towards anti-ubiquitin and M13KO7 and the effect of phage preincubation

#### Blocking effects of PTM buffer and E. coli lysate

Optimizations were done step-wise to investigate the binding capacity of the antibody towards the antigen in various blocking agents and target-unrelated proteins. Initially, a one-step affinity selection was performed separately with αUbi and M13KO7 against 10 µg of purified rUbi in the presence of various blocking agents. Eight microwells were coated with 100 μL of purified rUbi in PBS buffer overnight at 4 °C. The wells were washed three times with 0.1% (v/v) Tween-20 (PBS-T) and blocked with 300 μL of PTM blocking buffer (2% (w/v) skimmed milk in PBS-T) for 1 h with 600 rpm agitation at room temperature (RT). Simultaneously, the αUbi phage (10^10^ cfu/mL) and M13KO7 (10^11^ cfu/mL) were blocked separately with 4 sets of blocking agents in a total volume of 100 μL: i. PBS buffer, ii. 2% PTM, iii. *E. coli* lysate and iv. 2% PTM and *E. coli* lysate. The incubation took place for 1 h at RT, with gentle agitation at 600 rpm. Then, the antigen-coated wells were washed three times with PBS-T and the blocked phages were transferred into the wells accordingly. The binding took place at RT for 2 h at 600 rpm. The phage mixtures were collected at the end of incubation as ‘unbound phages’ and the wells were washed three times with PBS-T. The bound phages were eluted by enzymatic digestion with 100 μL of Trypsin (10 μg/mL in PBS buffer) for 30 min at 37 °C, static. Serial dilutions were performed for both unbound and eluted phages. The serially diluted phages were allowed to infect 200 μL of exponentially growing TG1 culture (OD_600nm_ = 0.5) at 37 °C for 30 min, static. The phage titres were determined by 10 μL spots on ampicillin-glucose-2YT agar and kanamycin-2YT agar plates. The titre data was collected as ‘Blocked phage’ and ‘Eluted phage’.

Next, biopanning simulations were carried out using a mixture of αUbi (10^10^ cfu/mL) and M13KO7 (10^11^ cfu/mL) against purified and crude rUbi in similar conditions. Preparations for antigen-coated wells and PTM-preblocked wells were similar as above. The mixture of αUbi and M13KO7 phages was blocked in 4 sets of blocking agents in a total volume of 100 μL: i. PBS buffer, ii. 2% PTM, iii. *E. coli* lysate and iv. 2% PTM and *E. coli* lysate. The blocking of phages took place for 1 h with 600 rpm agitation at RT and subsequently transferred into antigen-coated wells for binding. Phage titre data for unbound phage and eluted phage was determined. For biopanning simulation against crude rUbi, 10 μg of crude rUbi was coated onto microwells in PBS buffer. The total protein concentration of crude rUbi was measured with Nanodrop 2000 (Thermo Fischer Scientific, MA, USA). The procedures were similar as above.

#### Effect of phage preincubation

The second stage of optimization was performed with preincubation of phage prior to affinity selection against target antigen. Then, 100 μL of purified rUbi (10 μg) in PBS buffer was coated onto 8 microwells overnight at 4 °C for both αUbi and M13KO7 sample sets. Concurrently, the same number of wells were blocked with 300 μL *E. coli* lysate at 4 °C overnight. The antigen-coated wells and lysate preblocked wells were subjected to three times PBS-T washing. The wells were then blocked with 300 μL PTM blocking buffer for 1 h at RT, 600 rpm. The lysate/PTM-coated wells were washed three times with PBS-T and used for phage blocking. αUbi and M13KO7 were incubated with various blocking agents in lysate/PTM-coated wells, separately. Then, the phages were transferred into antigen-coated wells for binding to occur. The phage titres for both ‘Blocked phage’ and “Eluted phage’ were determined. For biopanning simulations, a mixture of αUbi and M13KO7 was preincubated prior to affinity selection against purified and crude rUbi. The rescued phages from biopanning simulations against crude rUbi for both optimizations were packaged and amplified. The phages were subjected to phage ELISA assay to assess the binding performance in the presence of various blocking agents. The detailed workflow is included in the Supplementary Methods 1–7.

### Construction of human naïve kappa light chain scFv phagemid library

A total of 52 cDNA of healthy human donors from three local ethnic groups (Malay, Chinese and Indian) was used as the starting material for library generation. Collection of human blood samples, total lymphocytes isolation, preparation of cDNA and library cloning were performed according to Lim, *et al*.^61^ with slight modifications. Sample collection was performed in accordance with the human ethical approval from the Human Ethics Committee of Universiti Sains Malaysia. All donors were informed about the project and gave their informed consent. All donors are healthy individuals with no known infections and have been physically healthy 3 months before collection. Detailed protocol for the library generation is described in Supplementary Method 8.

### ‘*Yin-Yang’* biopanning of human antibody scFv phage library against crude rMERS-NP

The affinity selection process was separated into 2 steps. The first step was carried out to capture the non-specific phages that are present in the phage library preparation. The second step was biopanning with ‘filtered’ phages from the previous steps against the target antigen.

A total of 0.2 mg of crude rMERS-NP in 100 µL of PBS buffer was coated to the surface of the microplate wells at 4 °C for 15 h. Simultaneously, an immunotube with high binding surface was blocked with equal volumes of *E. coli* lysate (generated from *E. coli* C41 (DE3) strain with empty pRSET-BH6) and 5% PTM at 4 °C, rotating for 15 h. All interval washes were performed 3 times with PBS-T. The antigen-coated well was blocked with 300 µL of 5% PTM buffer for 1 h, 600 rpm at RT. *‘Ying’* affinity selection was done in the immunotube with preincubation of library phage (100 µL of library phage at 10^11^ cfu/mL, 50 µL of 5% PTM and 50 µL *E. coli* lysate) for 1 h, rotating at RT. Then, the content in the immunotube was transferred into the well and incubated for 2 h, 600 rpm at 37 °C for *‘Yang’* affinity selection. At the end of binding, the wells were washed with 1% PBS-T (PBS buffer with 1% (v/v) Tween 20) for 10 times and 25 times for the subsequent rounds of biopanning. The bound phages were eluted with 100 µL of trypsin for 30 min at 37 °C. *E. coli* TG1 culture was infected with trypsin-eluted phage particles after first round of biopanning. The phage-infected TG1 cells were propagated and the phage particles were packaged and used for the second round of biopanning.

### Polyclonal antibody phage ELISA

The trypsin-eluted phages from two biopanning rounds were amplified and packaged. Two wells were coated with 0.2 mg of crude rMERS-NP while concurrently, another two wells were blocked with 5% PTM as background control. The enriched phages were blocked with equal volumes of 5% PTM and lysate in lysate/PTM-blocked immunotubes as described. Then, the phage mixtures were incubated in antigen-coated and background wells for 2 h at similar biopanning conditions. Anti-M13-horseradish peroxidase (HRP) was used to detect the bound phages for 1 h at RT, 600 rpm and 2,2′-Azino-bis(3-ethylbenzothiozoline-6-sulfonic acid) diammonium salt (ABTS) was used to develop a colorimetric signal. The enrichment pattern was determined at absorbance 405 nm.

### Monoclonal antibody phage ELISA and selection of anti-rMERS-NP clones

Monoclonal phage ELISA was carried out using the protocol according to Lim, *et al*.^61^ with slight modifications. Briefly, phage particles with the highest enrichment was subjected to TG1 infection and 92 single colonies were picked and cultured. The performance of the monoclones was assayed against 0.2 mg of crude rMERS-NP. 5% PTM was used as the sole blocking agent and three times washing with PBS-T was used at all the interval washes. The monoclonal phage particles were transferred into antigen-coated ELISA microplate and incubated for 2 h at 37 °C, 600 rpm. Then, anti-M13 HRP was introduced to detect phage binding followed by ABTS developing solution. The absorbance was measured at OD_405nm_ within 30 min. The detailed protocol is outlined in Supplementary Method 9.

### DNA sequencing

The positive clones from the monoclonal ELISA were cultured at 37 °C and 200 rpm for 15 h. The cell pellets were collected and plasmid extraction was performed using QIAprep Spin Miniprep Kit (Qiagen, Germany). The purified plasmids were sent for sequencing (FIRST Base Laboratories Sdn Bhd, Malaysia). The sequencing results were then analysed by IMGT/V-QUEST bioinformatics tool available at IMGT^®^, the International ImMunoGeneTics information system^®^ ^62,63^.

### Cloning of antibody genes into expression vector, antibody expression and purification

The A4scFv, C8scFv and G12scFv DNA were amplified from their respective pLABEL vectors and cloned into *E. coli* expression vector pRSET-BH6 at restriction sites *Nco*I and *Not*I (NEB, MA, USA). The resulting vectors were named pRSET-BH6 A4scFv, pRSET-BH6 C8scFv and pRSET-BH6 G12scFv. Expression of A4, C8 and G12 was performed with SHuffle^®^ T7 with pRARE3 for *in vivo* biotinylation. The expression condition for biotinylated A4, C8 and G12 was optimized to 24 °C, 140 rpm for 20 h with IPTG induction at OD_600nm_ 0.8 at a final concentration of 0.8 mM and 50 μM d-biotin. The inclusion bodies of biotinylated A4, C8 and G12 were solubilized using 8 M urea buffer, pH 8 (8 M Urea, 0.1 M NaH_2_PO_4_, 0.01 M Tris). The urea crude fractions were subjected to protein purification using Ni-NTA Agarose gravity column (Qiagen, Germany) at denaturing conditions. Initially, the column was equilibrated with 8 M urea buffer at pH 8 followed by sample loading. Then, the column was subjected to washing with 10 mL of 8 M urea buffer (pH 8). Step elutions were performed by 8 M urea buffer at pH 6.3, 5.9 and lastly 4.5, 2 mL elution fractions were collected stepwise. The protein fractions were analysed on SDS-PAGE. The last 5 fractions from A4, C8 and G12 were combined and subjected to buffer exchange using Amicon^®^ Ultra 0.5 mL Centrifugal Filters (Merck, Darmstadt, Germany). The antibody fragments were dissolved in 1X PBS buffer (pH 7.4). Western blotting was then performed using streptavidin-HRP (1:5000) (Thermo Scientific, MA, USA) to detect and confirm the respective antibody fragments.

### Antigen binding ELISA

The purified rMERS-NP (fused with His_6_-tag only) fractions, purified rGFP (fused with His_6_-tag only) fractions and purified antibody fragments were quantified (detailed in Supplementary Method 10). A serial dilution of purified rMERS-NP(His_6_) (μg: 1, 5, 10, 25, 50, 150) was coated onto microwells in PBS buffer with a total volume of 100 μL at 4 °C overnight. All interval washes were performed 3 times with PBS-T. The antigen-coated wells were blocked with 2% PTM blocking buffer for 1 h at RT, 600 rpm. The respective antibody fragments (A4, C8 and G12) were resuspended in PBS buffer, with each 100 μL aliquots representing 10 μg. Post-blocking, the wells were washed and each well was added with 100 μL of antibody fragments (10 μg). Additionally, 3 empty wells were also added 100 μL of antibody fragments as negative control wells. To check the cross-reactivity of antibodies towards His_6_-tagged proteins, 3 wells were coated 10 μg of rGFP(His_6_) and subsequently allowed to incubate with 100 μL of antibody fragments (10 μg). The antigen-antibody binding took place at 600 rpm, 37 °C for 2 h. Then, 100 μL of streptavidin-HRP (diluted at 1:5000 in PTM blocking buffer) was added into each well and incubated for 1 h at RT, 600 rpm to detect antigen-antibody binding. Lastly, 100 μL of ABTS developing solution was added after washing. The absorbance was measured at OD_405nm_ within 1 h.

## Supplementary information

Supplementary Info
