# Supplementary material for: Development of a Phage Display Panning Strategy Utilizing Crude Antigens: Isolation of MERS-CoV Nucleoprotein human antibodies
Source: Sci Rep. 2019 Apr 15;9:6088. doi: 10.1038/s41598-019-42628-6 (PMC6465254; doi:10.1038/s41598-019-42628-6)
Supplement: Supplementary file 1 — Supplementary Info [file 41598_2019_42628_MOESM1_ESM.docx]

**SUPPLEMENTARY INFORMATION**

**Development of a Phage Display Panning Strategy Utilizing Crude Antigens: Isolation of MERS-CoV Nucleoprotein human antibodies**

**Chia Chiu Lim^1^, Patrick CY Woo^2^ and Theam Soon Lim^1,3,*^**

^1^ Institute for Research in Molecular Medicine, Universiti Sains Malaysia, 11800 Penang, Malaysia

^2^ Department of Microbiology, The University of Hong Kong, Hong Kong

^3^ Analytical Biochemistry Research Centre, Universiti Sains Malaysia, 11800 Penang, Malaysia

* Tel: +604-653-4852, Fax: +604-653-4803, Email: [theamsoon@usm.my](mailto:theamsoon@usm.my)

**RESULTS**

**Supplementary result 1**

***‘Yin-Yang’* Optimization 2: Biopanning simulation of anti-ubiquitin and M13KO7 against rUbi in the presence of PTM and *E. coli* lysate**


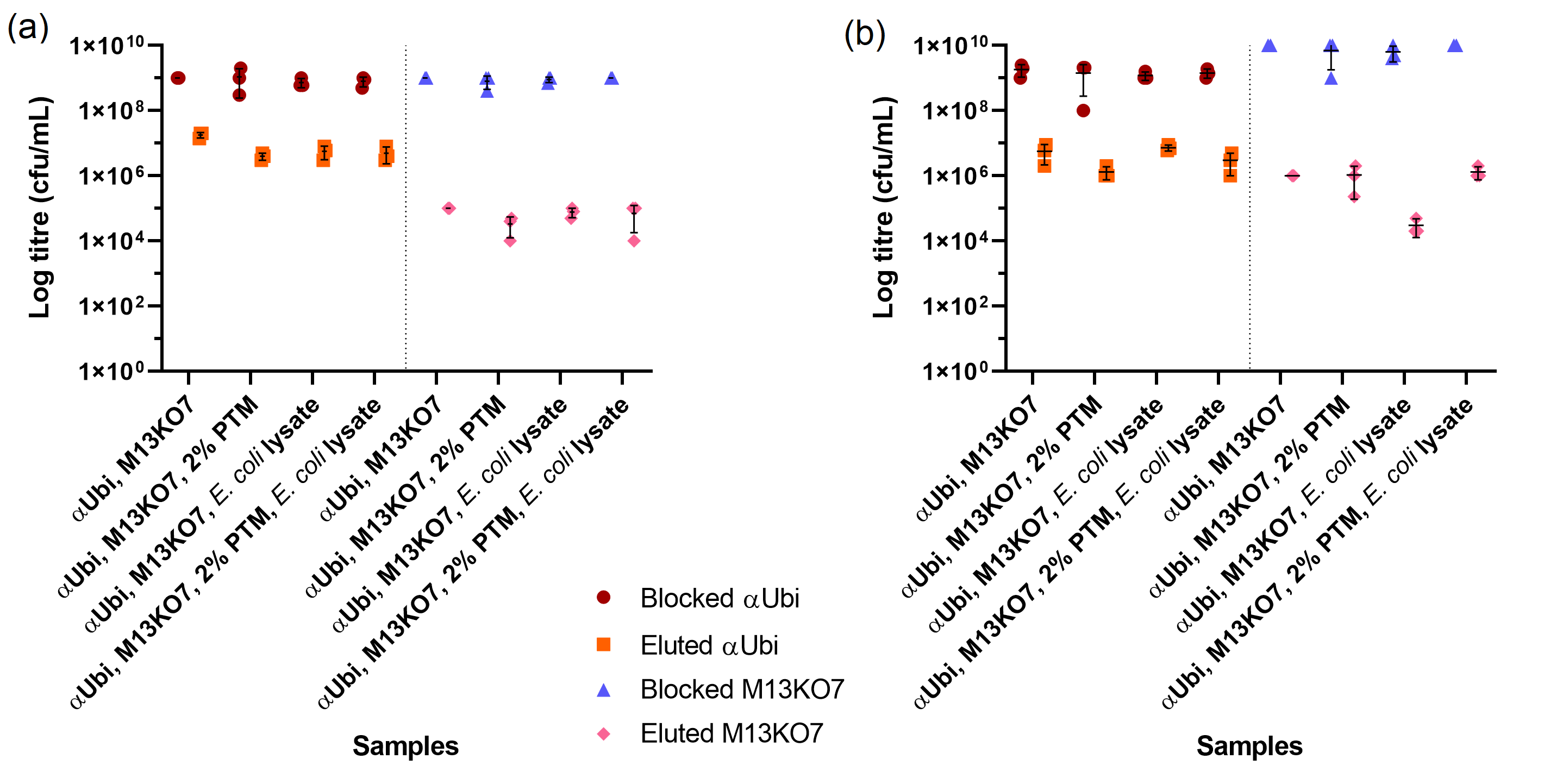


**Fig. S1.** Phage titres of biopanning simulation with a mixture of αUbi and M13KO7 against purified rUbi (a) and crude rUbi (b) in the presence of PTM buffer and lysate were collected in triplicates. Values were calculated and represented as mean ± s.d. (n=3) as shown in the scatter dot plot.

**Supplementary result 2**

***‘Yin-Yang’* Optimization 2A: Biopanning simulation of anti-ubiquitin and M13KO7 against rUbi upon lysate preblocking and phage preincubation**


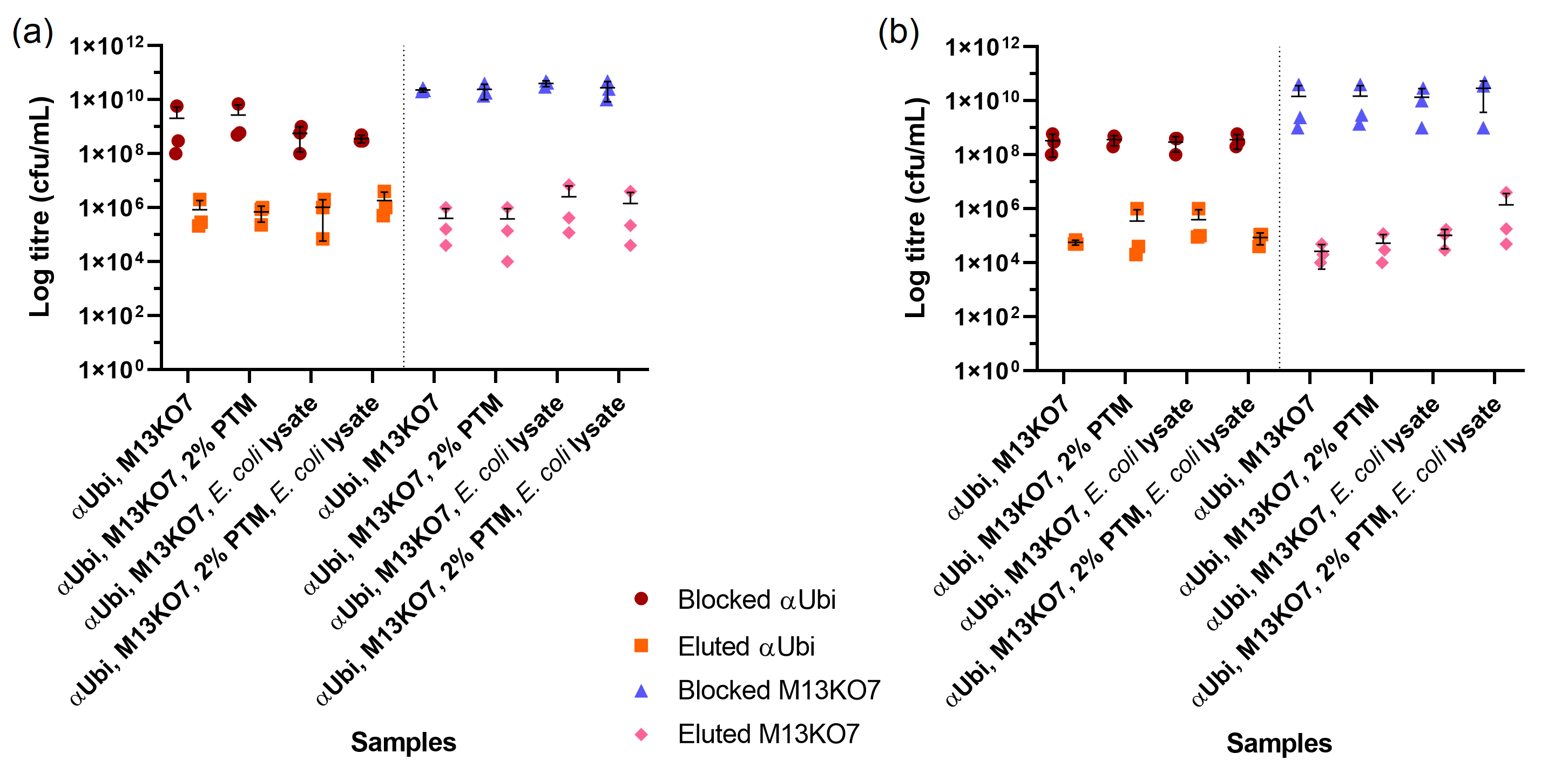


**Fig. S2.** Phage titres of ‘*Yin-Yang*’ biopanning simulation with a mixture of αUbi and M13KO7 against purified rUbi (a) and crude rUbi (b). The simulation was conducted in triplicates and the mean ± s.d. (n=3) was shown in the scatter dot plot.

**Supplementary result 3**

**Human naive kappa light chain scFv phagemid library construction**


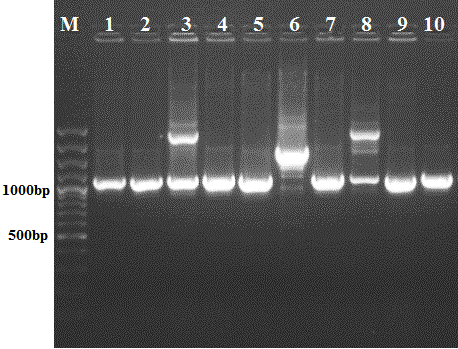


**Fig. S3.** Colony PCR of 10 randomly picked colonies from the ScFv antibody library. M is 100 bp DNA ladder; lane 1-10 shows the randomly selected monoclones from the naïve antibody library with the expected band size of 1100 bp.

**Supplementary result 4**

***‘Ying-Yang’* biopanning and polyclonal ELISA**





**Fig. S4.** Results of polyclonal phage ELISA assay against crude rMERS-NP after removal of background. Standard deviations were calculated from triplicates (n=3).

**Supplementary result 5**

**
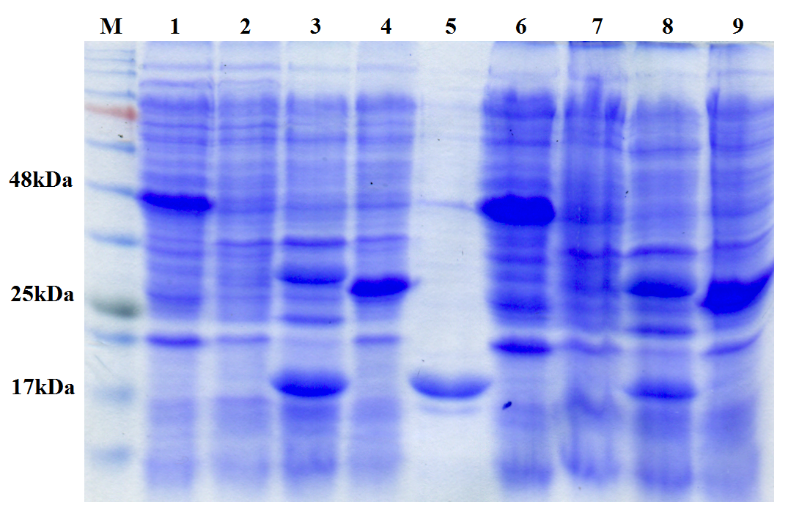
**

**Fig. S5.** Coomassie stained SDS-PAGE gel of rMERS-NP, rUbi and *E. coli* lysates. M indicates BLUelf prestained protein ladder (GeneDireX Inc.); lane 1 and 6, crude rMERS-NP; lane 2 and 7, *E. coli* C41 lysate; Lane 3 and 8, crude rUbi; Lane 4 and 9, *E. coli* BL21 with pRARE3 lysate; lane 5, purified rUbi. 10 µL of samples were loaded into lane 1-5 while 20 µL of samples for lane 6-9.

**Supplementary result 6**

**
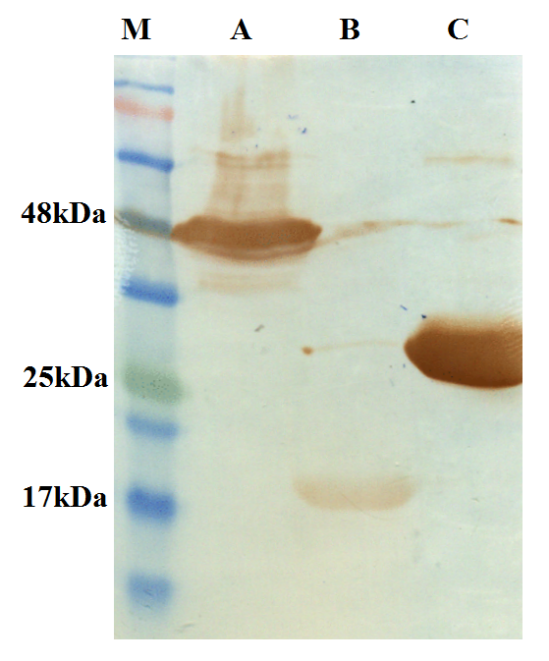
**

**Fig. S6.** Western blotting of rMERS-NP and purified rUbi. M indicates BLUelf prestained protein ladder (GeneDireX Inc.); lane A, crude rMERS-NP; lane B, purified rUbi and lane C, positive control. Sample loading was 15 µL and detected via ^1^_6_-tagged system.

**Supplementary result 7**


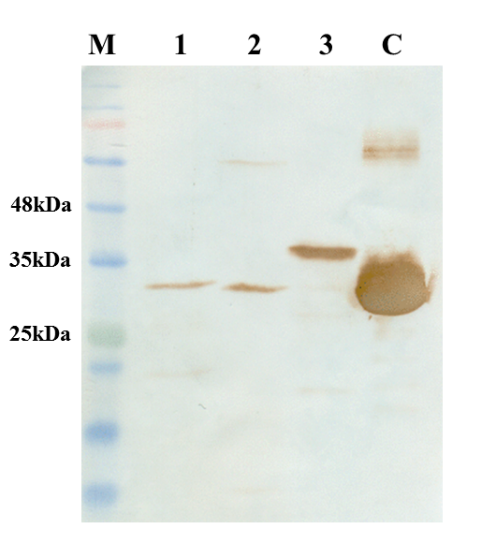


**Fig. S7.** Western blotting of antibody fragments rA4scFv, rG12scFv and rC8scFv. M Opti-Protein XL Marker prestained protein ladder (Abm Inc.); lane 1, purified rA4scFv; lane 2, purified rG12scFv; lane 3, purified rC8scFv and lane C, positive control. Sample loading was 15 µL and detected via Streptavidin HRP.

**Supplementary result 8**

**Preparation of rMERS-NP(His_6_) and reGFP(His_6_)**

rMERS-NP(His_6_) and reGFP(His_6_) were expressed, extracted and purified. Western Blotting using His_6_-tagged detection system was performed and showed specific band size of ~48 kDa for rMERS-NP(His_6_) and ~31 kDa for reGFP(His_6_) with good yield and purity in **Fig. S8**.


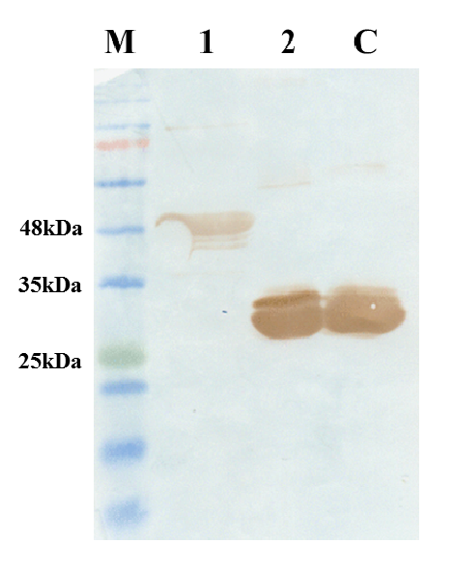


**Fig. S8.** Western blotting of antibody fragments rA4scFv, rG12scFv and rC8scFv. M Opti-Protein XL Marker prestained protein ladder (Abm Inc.); lane 1, purified rMERS-NP(His_6_); lane 2, purified reGFP(His_6_); lane C, positive control. Sample loading was 15 µL and detected via His_6_-tagged system.

**Table S1** Enrichment of phage from each rounds of *‘Yin-Yang’* biopanning.

|  | Input (CFU) | Output (CFU) | Ratio (Input/Output) |
| --- | --- | --- | --- |
| Round 1 | 1 x 10^11^ | 1 x 10^6^ | 1 x 10^-5^ |
| Round 2 | 4 x 10^10^ | 1 x 10^6^ | 2.5 x 10^-5^ |

**Methods**

**Supplementary method 1**

**Optimization 1: Blocking effects of PTM buffer and *E. coli* lysate towards anti-ubiquitin and M13KO7**

To investigate the binding effect of antibody towards antigen in the presence of blocking agent and miscellaneous proteins, a one-step affinity selection was performed separately with αUbi and M13KO7. 10 µg of purified rUbi was coated to the surface of high protein absorption microplate for overnight at 4 °C. The wells were washed three times with 0.1% (v/v) Tween 20 (PBS-T) and blocked with 300 µL of PTM blocking buffer (2% (w/v) skimmed milk in PBS-T) for 1 h with 600 rpm agitation at room temperature (RT). Simultaneously, the αUbi phage (10^10^ cfu/mL) and M13KO7 (10^11^ cfu/mL) were blocked separately with different sets of blocking agents in total volume of 100 µl: i. PBS, ii. 2% PTM, iii. *E. coli* lysate and iv. 2% PTM and *E. coli* lysate. Then, the antigen-coated wells were washed three times with PBS-T. The blocked phages were transferred into the wells accordingly and incubation took place at RT for 2 h at 600 rpm. The phage mixtures were collected at the end of binding as 'unbound phages' and the wells were washed three times with PBS-T. The bound phages were eluted by enzymatic method with 100 µL of Trypsin (10 µg/mL in PBS) for 30 min at 37 °C. Serial dilutions were performed for both unbound phages and eluted phages. The titres for both unbound phages and eluted phages were determined by infecting an exponentially growing TG1 culture (OD_600nm_= 0.5) for 30 min at 37 °C, static. The titres for both phages were spotted on ampicillin-glucose-2YT and kanamycin-2YT agar plates. The titre data was collected as 'Before elution' for unbound phages and 'After elution' for bound phages.

**Supplementary method 2**

**Optimization 2: Biopanning simulation of anti-ubiquitin and M13KO7 against rUbi in the presence of PTM and *E. coli* lysate**

A single biopanning round against rUbi was simulated with a mixture of αUbi phage (10^10^ cfu/mL) and M13KO7 (10^11^ cfu/mL) to determine the binding capacity of antibody towards antigen in the presence of blocking agents and complex proteins. 10 µg of purified rUbi was coated to the surface of high protein absorption microplate for overnight at 4 °C. The wells were washed three times with 0.1% (v/v) Tween 20 (PBS-T) and blocked with 300 µL of PTM blocking buffer (2% (w/v) skimmed milk in PBS-T) for 1 h, 600 rpm at RT. Simultaneously, the mixture of αUbi phage and M13KO7 were blocked separately with different sets of blocking agents: i. PBS, ii. 2% PTM, iii. *E. coli* lysate and iv. 2% PTM and *E. coli* lysate. Then, the antigen-coated wells were washed three times with PBS-T. The blocked phages were transferred into the wells accordingly and incubation took place at room temperature for 2 h at 600 rpm. At the end of binding, the phage mixtures were collected as 'unbound phages' and the wells were washed three times with PBS-T. The bound phages were eluted by enzymatic method with 100 µL of Trypsin for 30 min at 37 °C. Serial dilutions were performed for both unbound phages and eluted phages. The titres for both unbound phages and eluted phages were determined by infecting an exponentially growing TG1 culture (OD_600nm_= 0.5) for 30 min at 37 °C, static. The titres for both phages were spotted on ampicillin-glucose-2YT and kanamycin-2YT agar plates. The titre data was collected as 'Before elution' for unbound phages and 'After elution' for bound phages.

**Supplementary method 3**

**Optimization 3: Biopanning simulation of anti-ubiquitin and M13KO7 against crude rUbi in the presence of PTM and *E. coli* lysate**

A single biopanning round simulation was performed with a mixture of αUbi phage (10^10^ cfu/mL) and M13KO7 (10^11^ cfu/mL) against crude rUbi to determine the binding efficiency of antibody to crude antigen in the complex proteins microenvironment with the presence of blocking agents. The total protein concentration of crude rUbi was measured with Nanodrop 2000 (Thermo Fisher Scientific, MA, USA). Approximately 10 µg of crude rUbi was coated to the surface of high protein binding microplate for overnight at 4 °C. The biopanning simulation was performed as mentioned above in Supplementary method 2. The unbound phages were collected at the end of binding. Subsequently, the wells were washed three times with PBS-T. Trypsin elution was performed at 37°C for 30 min. Both phages were serially diluted and the titres for both unbound and eluted phages were determined by infecting an exponentially grown TG1 (OD_600nm_= 0.5) culture for 30 min at 37 °C, static. The titres for both phages were spotted on ampicillin-glucose-2YT and kanamycin-2YT agar plates. The titre data was collected as 'Before elution' for unbound phages and 'After elution' for bound phages.

The remaining eluted phages were rescued by infecting an exponentially grown TG1 (OD_600nm_=0.5) culture, 200 µL for 30 min at 37 °C, static. 30 µL of 10X ampicillin-glucose (ampicillin final concentration 100 µg/mL and glucose final concentration 2% (v/v)) was then added and grown at 37 °C, 800 rpm for overnight. A total volume of 20 µL overnight culture of phagemid bearing TG1 was inoculated into 180 µL 2YT broth, supplemented with 20 µL 10X ampicillin-glucose. The culture was further grown for 2 h at 37 °C, 800 rpm. The cells were co-infected with M13KO7 helper phage (10^7^ cfu/mL) for 30 min at 37 °C, static. The cells were centrifuged and the bacterial pellet was collected and resuspended in 270 µL 2YT media, supplemented with 30 µL 10X ampicillin-kanamycin (ampicillin final concentration 100 µg/mL and kanamycin final concentration 60 µg/mL) and 0.1% (v/v) glucose. Packaging and amplification of phage were done by culturing the cells at 30 °C, 800 rpm for overnight. The culture was then centrifuged and the phage containing supernatants were collected and stored in 4 °C.

**Supplementary method 4**

**Optimization 1A: Binding efficiency of anti-ubiquitin and M13KO7 against rUbi upon lysate preblocking and phage preincubation**

A one step affinity selection was performed to investigate the binding effect of antibody bearing phage towards antigen upon preblocking with lysate followed by preincubation of phage. 10 µg of purified rUbi was coated onto 8 wells for both sample sets (αUbi and M13KO7) at 4 °C for overnight. Also, same number of wells were blocked with 300 µL *E. coli* lysate at 4 °C for overnight. After incubation overnight, the antigen-coated wells and preblocked wells were washed with PBS-T for three times. The wells were blocked with 300 µL of PTM buffer at RT, 600 rpm for 1 h. The lysate/PTM-preblocked wells were washed with PBS-T for three times and were used to block the phages. The αUbi phage (10^10^ cfu/mL) and M13KO7 (10^11^ cfu/mL) were blocked separately with different sets of blocking agents in total volume of 100 µl: i. PBS, ii. 2% PTM, iii. *E. coli* lysate and iv. 2% PTM and *E. coli* lysate at RT, 600rpm for 1 h. The antigen-coated wells were washed three times with PBS-T and the blocked phages were added into the wells accordingly. Incubation took place at RT, 600 rpm for 2 h. At the end of binding, the phages were collected as 'Unbound phage' and proceeded to three times washing with PBS-T and trypsin elution as mentioned above. Both unbound phages and eluted phages were collected and serially diluted to determine the phage titres with TG1 infection and lastly the samples were spotted on agar plates supplemented with antibiotics (Refer Supplementary method 2).

**Supplementary method 5**

**Optimization 2A: Biopanning simulation of anti-ubiquitin and M13KO7 against rUbi upon lysate preblocking and phage preincubation**

A single biopanning round was simulated with a mixture of αUbi phage (10^10^ cfu/mL) and M13KO7 (10^11^ cfu/mL) to determine the binding efficiency of antibody towards antigen upon phage preincubation. 10 µg of purified rUbi in 100 µL of PBS buffer was coated onto 4 wells at 4 °C for overnight. Another 4 wells were blocked with 300 µL of *E. coli* lysate at 4 °C for overnight. The antigen-coated wells and the lysate-preblocked wells were washed with PBS-T for three times. All the wells were subjected to blocking with 300 µL of PTM buffer for 1 h, 600 rpm at RT. The lysate/PTM-preblocked wells were washed three times with PBS-T and the mixture of phages were blocked in the wells in total volume of 100 µL blocking agents i. PBS, ii. 2% PTM, iii. *E. coli* lysate and iv. 2% PTM and *E. coli* lysate at RT, 600 rpm for 1 h. The antigen-coated wells were subjected to three times washing with PBS-T. The phages were then transferred into the antigen-coated wells and incubated for 2 h, 600 rpm in RT. The phages were collected as 'Unbound phages' and the wells were washed three times with PBS-T. Trypsin elution was performed to elute bound phages. Both unbound and eluted phages were collected and diluted serially. Titres for both phages were determined.

**Supplementary method 6**

**Optimization 3A: Biopanning simulation of anti-ubiquitin and M13KO7 against crude rUbi upon lysate preblocking and phage preincubation**

A single round of biopanning was simulated with a mixture of anti-ubiquitin phage and M13KO7 against crude rUbi. 10 µg of crude rUbi in 100 µL of PBS buffer was coated on 4 wells. The steps were similar as mentioned in Supplementary method 5. The remaining eluted phages were rescued by infecting an exponentially growing TG1 (OD_600nm_= 0.5) culture for 30 min, 37 °C, static. The phage packaging and amplification steps were described in Supplementary method 2.

**Supplementary method 7**

**Phage ELISA assay for biopanning simulation of anti-ubiquitin and M13KO7 against crude rUbi**

4 wells were coated 100 µL of crude rUbi (10 µg) in PBS buffer for overnight at 4 °C. The microplate was washed with PBS-T for three times prior to blocking in 300 µL PTM buffer for 1 h, 600 rpm at RT. Another 4 wells without antigen coating were coated with 300 µL of PTM buffer concurrently as background control.

As for second biopanning simulation, the wells for phage preincubation were blocked with 300 µL of *E. coli* lysate at 4 °C for overnight. The antigen-coated wells and lysate-preblocked wells were washed three times with PBS-T and subsequently blocked with 300 µL of PTM buffer for 1 h, 600 rpm at RT. The wells were washed three times with PBS-T and 100 µl of the amplified phages were blocked with equal volumes of PTM buffer at RT, 600 rpm for 1 h.

The antigen-coated wells were washed three times with PBS-T prior to binding. 100 µL of the blocked phages were then transferred into both sample and background wells and incubated for 2 h at RT, 600rpm. Upon binding, the wells were washed three times with PBS-T followed by incubation with 100 µL of Anti-c-myc-HRP (1:5000) in PTM buffer for 1 h, 600 rpm at RT. The wells were then washed with PBS-T for three times. Lastly, 100 µL of the ABTS developing solution was added into the wells and incubated for 20 min, 600 rpm at dark. The absorbance was measured at OD_405nm_ within 30 min with Thermo Scientific Multiskan Spectrum microplate reader.

**Supplementary method 8**

**Construction of human naïve kappa light chain scFv phagemid library**

- *Gene cloning*

A total of 52 cDNA of healthy human donors from three local ethnic groups (Malay, Chinese and Indian) was used as the starting material for library generation. Collection of human blood samples, total lymphocytes isolation and preparation of cDNA were performed according to Lim, et al. ^2^ Sample collection was performed in accordance with the human ethical approval from the Human Ethics Committee of Universiti Sains Malaysia. All donors were informed about the project and gave their informed consent. All donors are healthy individuals with no known infections and have been physically healthy 3 months before collection.

Antibody V-gene amplification of kappa light chain and IgM isotype derived heavy chain (V_H_) from each donor was amplified using the primer set previously reported Lim, et al. ^3^. The primers were synthesized by Intergrated DNA Technologies Pte. Ltd. (IDT), Singapore. The polymerase chain reaction (PCR) amplification and purification for both V_K_ and V_H_ genes were optimized and conducted according to protocols reported in Lim, et al. ^2^ and Rahumatullah, et al. ^4^. The PCR products were resolved by electrophoresis in 1% (w/v) agarose gel and purified using QIAquick gel extraction kit (Qiagen). The templates from each donor were kept in -20 °C for second amplification. Second amplification was conducted to introduce restriction sites to template DNA from each donor for gene cloning. The PCR reactions was performed according to Lim, et al. ^2^ with replacement of 1 U DreamTaq DNA polymerase (Thermo Fisher Scientific) and amplification regime: initial denaturation (95 °C, 90 s), 20 cycles for denaturation (95 °C, 30 s), annealing (55 °C, 30 s) and extension (72 °C, 1 min) and final extension (72 °C, 5 min). At this point, all PCR products generated from all donors were resolved by electrophoresis in 1% (w/v) agarose gel and combined based on their respective V-gene families. Seven V_H_ gene families were purified and amplified with the primer set introducing the glycine-serine linker. Introduction of glycine-serine linker at 3'-end was done using the similar PCR amplification conditions with annealing temperatures optimized at 55-71 °C. The V_H_-glycine-serine products were resolved by electrophoresis in 1% agarose gel and purified using QIAquick gel extraction kit.

Restriction digestion of both V_H_-linker amplicons and V_K_ amplicons was carried out to generate overlapping overhangs for ligation. A 50 µL digestion mixture was prepared for 1 µg amplicon digestion using *Mlu*I restriction enzyme (NEB) supplemented with 1 x RE buffer and incubated at 37 °C for 12 h. The enzyme was inactivated by incubation for 20 min at 65 °C and purified using QIAquick PCR purification kit (Qiagen). The purified digested products were ligated to generate the full-length scFv gene construct. Ligation was performed in 1:1 ratio of both V_H_-linker and V_K_ amplicons for every ligation reaction. The ligation mixture consisted of 200 ng of both V_H_-linker and V_K_ purified products, 1 U T4 DNA Ligase (NEB) and 1 x T4 DNA Ligase buffer, ligation was performed at 4 °C for 12 h. The enzyme was inactivated at 65 °C for 20 min and the ligation product was subjected to ethanol precipitation with 0.1V of 3 M sodium acetate (pH 5.2) and 2.5V of chilled 100% ethanol, vortexed and left on bench for 20 min. This was followed by 20 min of centrifugation at 13,000 rpm and the supernatant was discarded. The pellet was washed with 100 µL of chilled 70% ethanol and centrifuged for 20 min at 13,000 rpm. Finally, the supernatant was discarded and the pellet was left to air dry on bench. The pellet was dissolved in autoclaved distilled water at desired volume. A final PCR amplification was conducted using complementary V-gene family primer pairs. 1 µL of precipitation product (approximately 100 ng) was used for each PCR reaction. The PCR amplification set up and conditions were the same as previously described. Lastly, the scFv gene products were resolved by electrophoresis in 1% (w/v) agarose gel and purified using QIAquick gel extraction kit.

- *TA cloning*

The generation of the naïve human phagemid library used TA cloning methodology to clone the PCR assembled scFv gene into an intermediate plasmid before cloning into the desired phagemid vector. The method was carried out according to Rahumatullah, et al. ^4^ with slight modifications. The scFv genes were cloned using TOPO^®^ TA Cloning^®^ kit (Invitrogen) with few optimizations. Briefly, the freshly amplified scFv gene with 3'-A overhangs (amplification with DreamTaq polymerase generates amplicons with 3'-A overhangs) was purified and 50 ng of PCR product was ligated to 1 U of pCR^™^ 2.1-TOPO^®^ vector, supplemented with 1 µL of salt in a 6 µL reaction mixture. The ligation was conducted at RT for 30 min followed by 30 min incubation on ice. Next, ethanol precipitation was subjected to the ligation mixture as described previously. 1 µL of precipitation product was then transformed into 60 µl of One Shot™ TOP 10 Electrocomp™ *E. coli* (Thermo Fisher Scientific) on ice by electroporation using a 0.1 cm cuvette and 1 ml of 2YT broth to resuspend the cells. The cells were left to grow at 37 °C, 750 rpm for 1 h and centrifuged. The pellet was resuspended with 1 mL new 2YT broth and plated on a 2YT agar plate (25 cm^2^) supplemented with 2% (v/v) glucose and 100 µg ml^-1^ ampicillin. The plate was incubated at 37 °C for 15 h. A total of 28 agar plates were plated. The colonies were then scraped off from the agar and combined into a single stock and left to grow at 37°C, 200 rpm for 20 min. Glycerol was added into the stock culture and distributed evenly into individual tubes and kept at -80 °C.

- *Diversity recovery*

500 mL of pLABEL (phagemid) vector culture and 1 L of TOPO library culture were prepared in 2YT media supplemented with 2% (v/v) glucose and 100 µg/mL ampicillin and left to grow at 37 °C and 200 rpm for 16 h. The cell pellets were harvested and subjected to plasmid extraction and purification using Plasmid *Plus* Maxi kit (Qiagen). Digestion was conducted with 1 µg of phagemid vector and 5 µg of plasmid DNA (TOPO library) by 1 U of *Nco*I RE and *Not*I RE (NEB), supplemented with 1 x RE buffer in a 50 µL digestion mixture. Digestion was performed at 37 °C for 4 h. The digested products were resolved by electrophoresis in 1% (w/v) agarose gel and purified using QIAquick gel extraction kit. Dephosphorylation of the purified digested plasmid was done using 1 U of Antarctic Phosphatase (NEB) and 1 x AP buffer in 30 µL reaction mixture and incubated for 1 h at 37 °C. Ligation was performed by introducing the scFv gene into pLABEL vector in 3:1 ratio. 130 ng of scFv inserts and 200 ng of pLABEL plasmid DNA were ligated by 1 U of T4 DNA Ligase and 1 x T4 DNA Ligase buffer at 16 °C for 12 h. The ligated product was heat inactivated and subjected to ethanol precipitation as described. Then 1 µL of the precipitation product was transformed into 60 µL XL1-Blue MRF' electrocompetent cells (Stratagene) on ice. Transformation was performed as described previously and plated on 25 agar plates.

The library size was estimated by taking 10 µL from the stock culture and diluted with 90 µL of 2YT media for dilution factor of 10^-2^. Serial dilution was performed until a final dilution of 10^-10^. A total of 100 µL of each dilution was plated on 2YT agar plate (100 x 15 mm) and incubated at 37 °C for 15 h. The number of colonies on each agar plate was determine and extrapolated for library size estimation. Single colonies were picked and colony PCR was performed. A total of 10 random clones were sent for DNA sequencing (FIRST Base Laboratories Sdn Bhd, Malaysia).

**Supplementary method 9**

**Monoclonal antibody phage ELISA and selection of anti-rMERS-NP clones**

Phage particles from the round with the highest enrichment were subjected to TG1 infection at 37 °C for 30 min. The cells were centrifuged and plated on 2YT agar plate supplemented with ampicillin and glucose. A total of 92 colonies were picked and grown in 2YT broth with 2% (v/v) glucose and 100 µg/mL ampicillin at 37 °C and 1400 rpm for 15 h in Nunc™ 96-well round bottom culture plate. On the plate, wells in positions H5 and H11 were cultured with a known clone (anti-Ubi scFv) as positive controls while H6 and H12 were left empty as negative controls. The next day, 20 µL of the 16 h culture was inoculated into 180 µL of 2YT broth with 2% (v/v) glucose and 100 µg/mL ampicillin at 37 °C, 1400 rpm for 2 h. Then 10 µL of M13KO7 helper phage (10^9^ cfu/mL) was added into each well to enable co-infection at 37 °C, 1 h, static. Then, the culture plate was centrifuged and the supernatant was discarded. The cells were resuspended with 200 µL 2YT broth with 0.1% (v/v) glucose, 100 µg/mL ampicillin and 60 µg/mL kanamycin and continued to grow at 30 °C and 1400 rpm for 15 h. The plate was centrifuged and the supernatant containing phage particles were collected for ELISA. A total of 0.2 mg of crude rMERS-NP was coated to the surface of high protein absorption 96 well microplates at 4 °C for 15 h. Wells in positions H5 and H11 were coated with rUbi while wells H6 and H12 were left empty. MAb antibody phage ELISA was performed by first blocking the ELISA plate with 5% PTM buffer for 1 h at RT, 600 rpm. Three times washing with PBS-T was used at all the interval washes. The phage particles were transferred to sample wells and background wells with equal volumes of 5% PTM buffer and incubated for 2 h at 37 °C, 600 rpm. Then 100 µL of anti-M13-HRP diluted in 5% PTM buffer (1:2500) was added to the ELISA plates and incubated for 1 h at RT, 600 rpm. Lastly, 100 µL ABTS developing solution was added to the ELISA plates and incubated in the dark for 30 min shaking. The absorbance was measured at OD_405nm_ within 30 min with an absorbance microtitre plate reader (Thermo Scientific Multiskan Spectrum).

**Supplementary method 10**

**Preparations of rMERS-NP(His_6_) and reGFP(His_6_) for antigen binding ELISA**

The recombinant plasmid pRSET-BH6 bearing MERS-NP gene were subjected to avidin tag removal to avoid *in vivo* biotinylation. The resulting vector, named as pRSET-H6 MERS-NP consists of rMERS-NP with only hexahistidine tag. The rMERS-NP was expressed using *E. coli* C41 (DE3) strain in similar conditions and followed by cytoplasmic extraction. The rMERS-NP was subjected to IMAC purification using Ni-NTA Agarose gravity column (Qiagen, Germany). The purified fractions were analysed on SDS-PAGE and followed by protein quantification. Meanwhile, the reGFP was expressed in *E. coli* BL21 (DE3) strain in similar conditions and the crude lysate was also subjected to IMAC purification.

**Reference list:**

1 McCafferty, J., Griffiths, A. D., Winter, G. & Chiswell, D. J. Phage antibodies: filamentous phage displaying antibody variable domains. *Nature* **348**, 552, doi:10.1038/348552a0 (1990).

2 Lim, B. N., Chin, C. F., Choong, Y. S., Ismail, A. & Lim, T. S. Generation of a naïve human single chain variable fragment (scFv) library for the identification of monoclonal scFv against Salmonella Typhi Hemolysin E antigen. *Toxicon* **117**, 94-101 (2016).

3 Lim, T. S. *et al.* V-gene amplification revisited–An optimised procedure for amplification of rearranged human antibody genes of different isotypes. *New biotechnology* **27**, 108-117 (2010).

4 Rahumatullah, A., Ahmad, A., Noordin, R. & Lim, T. S. Delineation of BmSXP antibody V-gene usage from a lymphatic filariasis based immune scFv antibody library. *Molecular immunology* **67**, 512-523 (2015).
